# Cholesterol-mediated inflammation activation in alveolar macrophages

**DOI:** 10.1186/s12915-025-02494-3

**Published:** 2025-12-22

**Authors:** Sonia Giambelluca, Matthias Ochs, Elena Lopez-Rodriguez

**Affiliations:** 1 Institute of Functional Anatomy, Charité—Univeristätsmedizin, Berlin, Germany; 2 German Center for Lung Research (DZL), Berlin, Germany

**Keywords:** Cholesterol crystal, NLRP3 inflammasome, Liposome, RCT pathway, Interleukin-1β, ASC speck, Primary alveolar macrophage

## Abstract

**Background:**

The role of cholesterol- and cholesterol crystal-mediated inflammation has been extensively investigated in circulating macrophages in the context of cardiovascular diseases while little is known about its contribution to lung diseases. However, lipid-laden alveolar macrophages and crystal-like structures have been reported in lungs of different human and animal models of lung diseases. In this study, we address the hypothesis that a mechanism of inflammasome activation, due to altered cholesterol metabolism, may occur also in alveolar macrophages.

**Results:**

We tested the effect of soluble (cholesterol-enriched liposomes) and crystalized cholesterol exposure in a cell model of macrophages and in primary murine alveolar macrophages. Both soluble and crystalized cholesterol can be taken up by macrophages, mainly by phagocytosis for the crystals. Prolonged exposure to both forms of cholesterol leads to intracellular cholesterol accumulation in cytoplasmic lipid droplets and to foam cell formation in a time-dependent manner. However, in unprimed alveolar macrophages, immunofluorescence detection of the NLRP3 inflammasome and analysis of inflammatory cytokines showed that only cholesterol crystals stimulate the assembly of the inflammasome in speck and release of IL-18, indicating the sterile activation of the inflammasome. The role of NLRP3 was confirmed by chemical inhibition of the NLRP3 inflammasome in vitro and by validation in macrophages from NLRP3-deficient mice.

**Conclusions:**

In alveolar macrophages, cholesterol crystals, but not soluble cholesterol, trigger the assembly and activation of the inflammasome, thus leading to the inflammasome-dependent release of IL-18. These results open new scenarios for the role of alveolar macrophages and of cholesterol-mediated inflammation in the lung.

**Supplementary Information:**

The online version contains supplementary material available at 10.1186/s12915-025-02494-3.

## Background

In the lung, alveolar macrophages (AM) play a critical role in tissue homeostasis and represent the first line of defense against pollutants and pathogenic microbes, activating the innate immune response [1]. Along with the host defense function, AM are partially responsible, together with alveolar epithelial type 2 cells (AT2C), for lung surfactant metabolism and specifically catabolism [2], even though the specific pathways are still not known. Indeed, the correct balance of lung surfactant lipid species, accounting for 90% of its composition, is strictly regulated and dependent on systemic metabolic status. Among lung surfactant lipids, cholesterol is the most abundant neutral lipid (8–10 wt.% or 14–20 mol.%) [3, 4] and regulates surfactant fluidity and function.

Excessive amounts of cholesterol are mainly taken up by AM and can be exported from the cell via the reverse cholesterol transport (RCT) pathway [5] or stored intracellularly in form of lipid droplets, leading to the formation of lipid-laden or foam cells. Indeed, disruption in surfactant lipid homeostasis leads to lipid-laden AM, a common pathological feature already observed in patients with chronic obstructive lung disease [6], silicosis [7], or pulmonary alveolar proteinosis [8]. Moreover, evidence of foam cells has been reported in different animal models of lung injury and disease, such as cigarette smoke exposure [9], silicosis [10], and bleomycin-induced lung fibrosis [11], the latter providing strong evidence for alternative activation of AM as an important signaling pathway in the pathogenesis of lung fibrosis [12]. In Abcg1^−/−^ mice, accumulation of intracellular lamellar bodies, cholesterol ester droplets, and cholesterol clefts has been reported in AT2C and AM [13]. Recently, accumulation of needle-shaped crystals has been observed in mouse lungs from different disease models. Though the chemical nature of these structures is still unclear, since they have previously been identified as Ym-1 crystals [12, 14] or cholesterol crystals (CC) [15]. Ruwisch and colleagues [16] reported that the presence of crystals in AM of surfactant protein C knockout (Sftpc^−/−^) mice occurred along with changes in cholesterol metabolism.

Foam cells and CC are a recognized characteristic feature in atherosclerotic lesions in which they trigger both a local and systemic inflammation [17, 18]. Indeed, excessive cholesterol accumulation results in the formation of crystals that engulfed by macrophages induce lysosomal damage and translocation of phagolysosomal content into the cytosol, activating the NOD-like receptor protein-3 (NLRP3) inflammasome [18]. In addition to CC phagocytosis, crystal nucleation also occurs intracellularly upon cholesterol ester [19], soluble acetylated [20], or oxidized low-density lipoprotein (oxLDL) recognition and internalization by macrophages via cell-surface receptors, such as CD36 [21]. Both CC and oxLDL promote inflammatory responses including neutrophil influx and NLRP3 inflammasome activation with subsequent release of pro-inflammatory cytokines in LPS-primed macrophages, thus playing an important role in the development of atherosclerosis pathogenesis and plaque progression [21–24].

AM express most of the proteins and receptors involved in the uptake and clearance of lipids in atherosclerotic plaques [7]. Moreover, AM are the first line of defense in silicosis, where the engulfment of crystalline silica leads to the formation of foam cells and subsequent secretion of pro-inflammatory and pro-fibrotic cytokines and chemokines, including IL-1β, IL-6, TNF-α, and monocyte chemoattractant protein-1 [25, 26]. However, the hypothesis of a cholesterol-mediated activation of AM to a pro-inflammatory phenotype has not been addressed yet.

With the present study, we aim to fill this gap and elucidate whether soluble cholesterol and/or CC uptake can promote foam cell formation and trigger the sterile production of pro-inflammatory markers in AM. For this purpose, we firstly tested our hypotheses in phorbol-12-myristate 13-acetate (PMA)-stimulated THP-1-derived macrophages [27], previously reported to be able to accumulate CC and activate NLRP3 inflammasome, when primed by high dose of PMA and treated with preformed CC [28]. However, a resting period after PMA was applied to exclude biases on the activation of NLRP3 inflammasome [29]. For the same reason, LPS priming was omitted and a sterile triggering of the inflammasome was rather investigated.

As a major novelty, the response to cholesterol-rich liposome was tested, to mimic the lipid condition to which AM are exposed in the lung during healthy and pathologic conditions.

The conditions set in THP-1-derived macrophages were then applied directly in mouse primary AM. The results could provide new insights into a possible role of macrophages and of cholesterol-mediated inflammation in the lung, opening the way to new therapeutic targets.

## Results and discussion

### Macrophages-like cells (MLC) uptake of cholesterol is dose-dependent

The capability of MLC to uptake cholesterol in form of CC or cholesterol-enriched liposomes was evaluated by fluorescence-activated cell sorting analysis FACS, after exposure to the stimuli for 12 h and 24 h in serum-free medium, in presence or absence of potential uptake inhibitors. The gating strategy applied is reported in supplementary material (Additional file 1: Fig. S1A). The setup of the FACS method was performed by using MLC treated with oxLDL-DyLight 488 alone as technical positive control, or with the CD36 inhibitor myricetin 25 µM 2 h prior oxLDL as inhibitor control (Additional file 1: Fig. S1B and C).

**Fig. 1:**
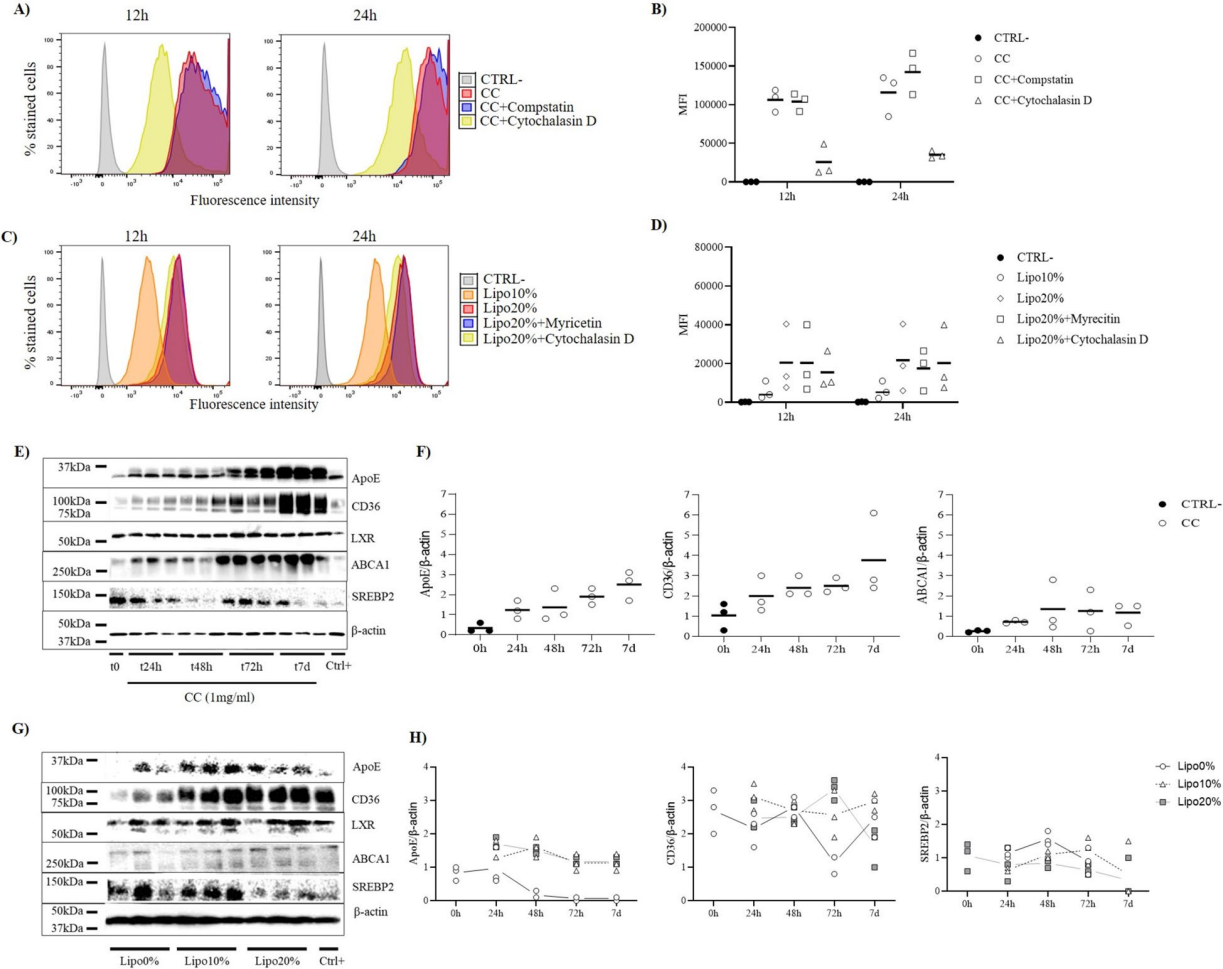
Assessment of the uptake and metabolism of CC and cholesterol-enriched liposomes in MLC. **A**, **B** FACS analysis of MLC untreated (CTRL −, gray) or treated with bodipy-labeled CC at 1 mg/ml for 12 h and 24 h alone (CC, red) or treated with compstatin (CC + compstatin, blue) at 20 µM 5 min or with cytochalasin D (CC + cytochalasin D, yellow) at 5 µM 10 min prior and during treatment with CC. **C**, **D** FACS analysis of MLC untreated (CTRL −, gray) or treated with 200 µg/ml bodipy-labeled cholesterol-enriched liposomes at 10% (Lipo10%, orange) and 20% (Lipo20%, red) cholesterol for 12 h and 24 h alone or treated with myricetin 25 µM 2 h (Lipo20% + myricetin, blue) or with cytochalasin D (Lipo20% + cytochalasin D, yellow) at 5 µM 10 min prior and during treatment with Lipo20%. **A**, **C** Representative histogram plots of y: percentage of stained cells normalized by mode vs x: fluorescence intensity; **B**, **D** mean fluorescence intensity (MFI) for the different treatment groups. **E** Representative results of western blot analyses for proteins of the RCT pathway and β-actin and **F** densitometry analysis of target proteins to the loading control (β-actin) in cell lysate of MLC incubated with CC 1 mg/ml for t 0 h, 24 h, 48 h, 72 h, or 7 days (7 d). **G** Representative results of western blot analyses for proteins of the RCT pathway and β-actin in cell lysate of MLC incubated with cholesterol-enriched liposomes 200 µg/ml at 0% (Lipo0%), 10% (Lipo10%), or 20% (Lipo20%) cholesterol for 72 h and **H** densitometry analysis of target proteins to the loading control (β-actin) in Lipo0%, Lipo10%, and Lipo20% at t 0 h, 24 h, 48 h, 72 h, or 7 d. The positive control (Ctrl +) for the validation of western blot antibodies was obtained by incubation of MLC with oxLDL 25 µg/ml for 48 h. Representative blots of the other time points are shown in Suppl Fig. 2A. Data are reported as mean of three independent experiments in different plates with three different cell passages and freshly prepared stimuli (*N* = 3). Individual data values are provided in an additional excel file (Additional file 2: Supporting data values)

For experiments with fluorescence-labeled CC, 100% of cells resulted fluorescence-positive at both time points after stimulus, indicating that MLC can uptake cholesterol in crystalline form. No difference in the percentage of positive cells was found between the two time points, and only a slight increase in the mean fluorescence intensity (MFI) was detected at 24 h compared to the 12 h treatment (Fig. 1A and B). The pretreatment of MLC with cytochalasin D or compstatin as potential inhibitors for the uptake did not lead to a reduction of the percentage of fluorescence-positive cells (> 98% in both inhibitor groups); however, the group treated with cytochalasin D showed a decrease in MFI at both 12 h and 24 h (Fig. 1A and B). Of note, for MLC treated with CC alone or with CC after pretreatment with compstatin, 10% and 15% of total events at 12 h and 24 h analyses, respectively, had off-the-chart intensity that could have affected the accuracy of the reported MFI values. Cytochalasin D is a widely used inhibitor of phagocytosis interfering with actin polymerization [30], already reported to block phagocytosis of osmium-stained *Escherichia coli* (*E. coli*) DH5-α cells in monocyte-derived macrophages at the chosen concentration [31]. Moreover, cytochalasin D inhibits the release of cytokines, namely IL-18, by microglia and ARPE-19 cells primed with LPS plus IL-1a and then stimulated with CC [32]. Although the uptake was not completely inhibited in our case, the decrease in MFI in the cytochalasin D group suggests that the uptake of CC may involve, as a non-exclusive mechanism, F-actin-mediated phagocytosis, according to previous reports [23, 28]. No changes in MFI were detected after treatment with fluorescence-labeled CC in the presence of compstatin, a potent and selective inhibitor of complement receptor 3 (CR3), at the chosen concentration, differently from what was previously proposed [33]. The discrepancy in the obtained results compared to the previous study may rely on the length of the exposure time, since Samstad and colleagues reported inhibition of 50–60% after 20-min incubation with CC, which decreased to a minimum after 1-h incubation.

Likewise, all the MLC treated with 200-µg/ml fluorescence-labeled cholesterol-enriched liposome at 10% (Lipo10%) and 20% (Lipo20%) of cholesterol were 100% fluorescence-positive, with MFI increasing in Lipo20% compared to the Lipo10% group (Fig. 1C and D). For liposome-treated MLC, no difference between treatment group and inhibitor groups, namely treated with myricetin (inhibitor for CD36) and cytochalasin D prior and during treatment with Lipo20%, was found neither in the percentage of fluorescence-positive cells nor in MFI (Fig. 1C and D), suggesting that another mechanism may mediate the cholesterol-enriched liposome uptake.

Taken together, these results indicate that MLC are able to uptake cholesterol from both CC and liposome in a dose-dependent manner. F-actin-mediated phagocytosis contributes to the uptake of CC.

### Metabolism of CC and cholesterol-enriched liposomes involves the reverse cholesterol transport (RCT) pathway

To evaluate if the internalized cholesterol is metabolized through the RCT pathway, the level of the main proteins of the pathway was determined. For MLC treated with CC, a time-dependent increase of apolipoprotein E (ApoE), CD36, and ATP-binding cassette transporter (ABCA1) was found (Fig. 1E and F). Similarly, higher levels of ApoE were found in MLC treated with cholesterol-enriched liposomes compared to cholesterol-free liposomes (Lipo0%) starting from 48 h after exposure, although no differences between Lipo10% and Lipo20% was found (Fig. 1H and Additional file 1: Fig. S2A). For the cholesterol transporter CD36, a clear dose-dependent effect of cholesterol was detected at 72 h, with higher levels of the protein in Lipo20% group compared to both Lipo0% and Lipo10% (Fig. 1G and H). The level of CD36 decreases at 7 days in the Lipo20% group, indicating a possible negative regulation of the protein following the time-dependent accumulation of cholesterol (Fig. 1H and Additional file 1: Fig. S2A). Accordingly, levels of sterol regulatory element-binding protein-2 (SREBP2), a transcription factor involved in cholesterol synthesis and import, decreased in Lipo20% compared to Lipo0% and Lipo10% at 48 h and 72 h after exposure (Fig. 1G and H and Additional file 1: Fig. S2A). Although the treatment with myricetin was not able to inhibit the CD36-mediated uptake of liposomes after short exposure (12–24 h), its role may be more prominent after longer exposure. Moreover, CD36 was already reported to be involved in foam cell formation and is crucial for nucleation of NLRP3 ligand inflammasome activation in sterile inflammation in macrophages [21]. On the other hand, ApoE is an apolipoprotein acting as transporter of extracellular cholesterol and reported to function as a concentration-dependent pulmonary danger signal that primes and activates the NLPR3 inflammasome in AM of asthmatic patients [34]. Moreover, ABCA1 increase, as found after CC treatment, represents the first step of the activation of the RCT and mediates the efflux of excessive cholesterol from the macrophages [35].

Taken together, these data suggest that metabolism of CC and cholesterol-enriched liposomes in MLC may occur through RCT pathway and point to ApoE and CD36 as key factors for the production of foam cells.

### Prolonged exposure to cholesterol leads to foam cell formation in MLC

**Fig. 2:**
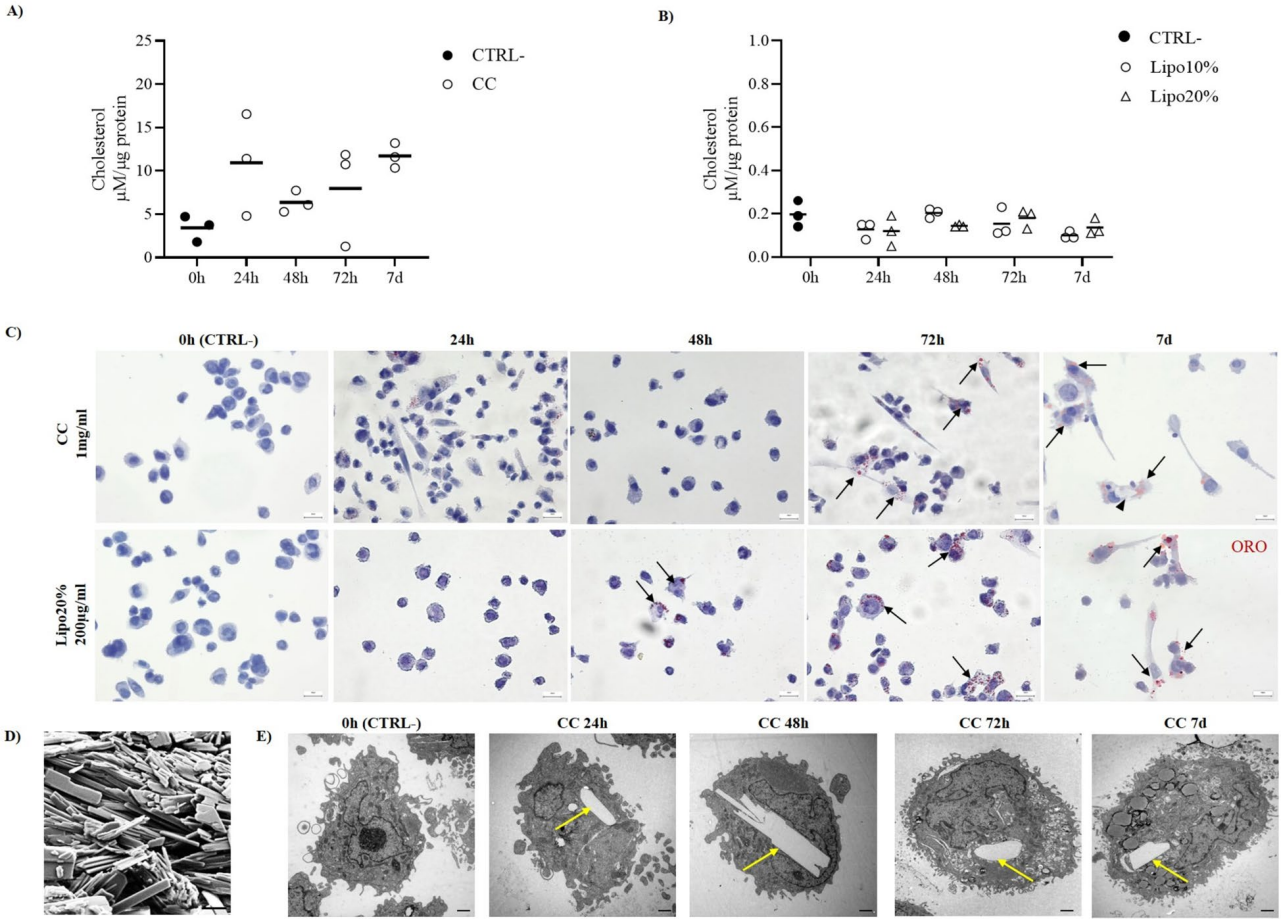
Foam cell formation and CC accumulation in MLC. Fluorometric cholesterol quantification corrected by µg of proteins in cell lysate of MLC treated with **A** cholesterol crystals (CC) at 1 mg/ml or **B** cholesterol-enriched liposomes 200 µg/ml at 10% (Lipo10%) or 20% cholesterol (Lipo20%) for 0 h, 24 h, 48 h, 72 h, or 7 days (7 d), 0 h served as negative control (CTRL −). Data are reported as mean of three independent experiments in different plates with three different cell passages and freshly prepared stimuli (*N* = 3). **C** Oil Red O (ORO) staining of MLC treated with CC 1 mg/ml (upper panels) or with cholesterol-enriched liposomes 200 µg/ml at 20% cholesterol (Lipo20%, lower panels) for 0 h (CTRL −), 24 h, 4 8 h, 72 h, or 7 d. Intracellular neutral lipid droplets are stained in red, cells are counterstained with Mayer’s hematoxylin (blue), and foam cells are indicated by black arrows. In the CC group, at 7 d a crystal (arrowhead) can be observed in the cytoplasm of an AM along with ORO-stained lipid droplets. Scale bar 20 μm. **D** Scanning electron microscopy (SEM) micrograph of CC. Scale bar 10 μm. **E** Representative transmission electron microscopy (TEM) micrographs of MLC treated with CC 1 mg/ml for 0 h (CTRL −), 24 h, 48 h, 72 h, or 7 d. Internalized CC are indicated by yellow arrows. All the micrographs are reported with the same scale (scale bar 1 μm). Individual data values are provided in an additional excel file (Additional file 2: Supporting data values)

To determine whether the treatment with CC and cholesterol-enriched liposomes at the chosen concentration causes intracellular accumulation of cholesterol, the cholesterol content of the cells was measured by a fluorometric assay, and the formation of foam cells was evaluated by Oil Red O (ORO) staining at different exposure times. The quantification of cholesterol showed a time-dependent increase in intracellular cholesterol in MLC treated with CC (Fig. 2A). MLC treated with oxLDL 50 µg/ml for 24 h was used as technical control for foam cell detection and showed accumulation of red Oil Red O (ORO)-stained cytoplasmic lipid droplets (Additional file 1: Fig. S2C). While untreated MLC did not show any foam cells, and only a few ORO-stained lipid droplets were detected at 24 h, accumulation of foam cells was detected starting from 72 h after CC treatment, indicating that long exposure to CC causes foam cell formation (Fig. 2C, upper panels).

MLC exposed to cholesterol-enriched liposomes showed no differences in cholesterol content among time points or between the Lipo10% and Lipo20% (Fig. 2B). The lack of discrimination among different groups may be due to the detection limit of the assay used. Indeed, the ORO-staining analysis confirmed that cholesterol accumulates intracellularly in lipid droplets and causes foam cell formation starting from 48 h after exposure in Lipo20% group (Fig. 2C, lower panels) and after 72-h exposure in Lipo10% group (Additional file 1: Fig. S2D). Of note, the difference in the 0 h negative control baseline between the experiments with CC and with the liposomes is due to technical reasons. Indeed, the stimulus was in both cases applied at 0 h before collection to simulate the bias in calculation due to the extracellular cholesterol that cannot be completely removed at sample collection, despite repeated PBS washes. The application of CC at time 0 h causes a mean increase in the cholesterol baseline of about 3 µM/µg protein in MLC (Additional file 1: Fig. S2B).

The shape and size of synthetic CC was evaluated by scanning electron microscopy (SEM), as shown in Fig. 2D. TEM analysis confirmed the presence of crystals with similar shape as the ones visualized by SEM in the cytoplasm of treated MLC at any of the tested time points (Fig. 2E, yellow arrows). On the other hand, the uptake of cholesterol-enriched liposomes at the chosen concentration did not cause intracellular cholesterol crystallization. Previous studies reported that crystal nucleation also occurs intracellularly in macrophages loaded with cholesterol ester [19], acetylated low-density lipoprotein (LDL) [20], or oxLDL [21]. Here, we decided to use liposomes enriched with non-esterified cholesterol, rather than the previously reported forms of cholesterol, to mimic surfactant composition to which the AM are exposed in the lung environment. The chosen concentration and/or the use of free cholesterol in liposomes may be the reason for the different result.

These data indicate that uptake of CC and cholesterol-enriched liposomes causes intracellular cholesterol accumulation in cytoplasmic lipid droplets, leading to foam cells in a time-dependent manner. The increase in the concentration of ApoE and CD36 along with the presence of foam cells at 72 h in both CC and liposome experiments confirms their essential role as transporters of cholesterol also in crystalline and liposome forms. As an effect of the activation of RCT, the removal of cholesterol from macrophage foam cells should occur. The efflux of cholesterol is mostly mediated by ABCA1 that in our study showed a time-dependent increase after CC treatment, although no clearance of foam cells was detected. However, the timing between the protein level increase and the disappearance of the foam cells may be longer than the time points explored here and/or could have been affected by an excess of cholesterol in the treatment that could have endorsed the formation of new foam cells.

### Uptake of cholesterol activates an inflammatory response

**Fig. 3:**
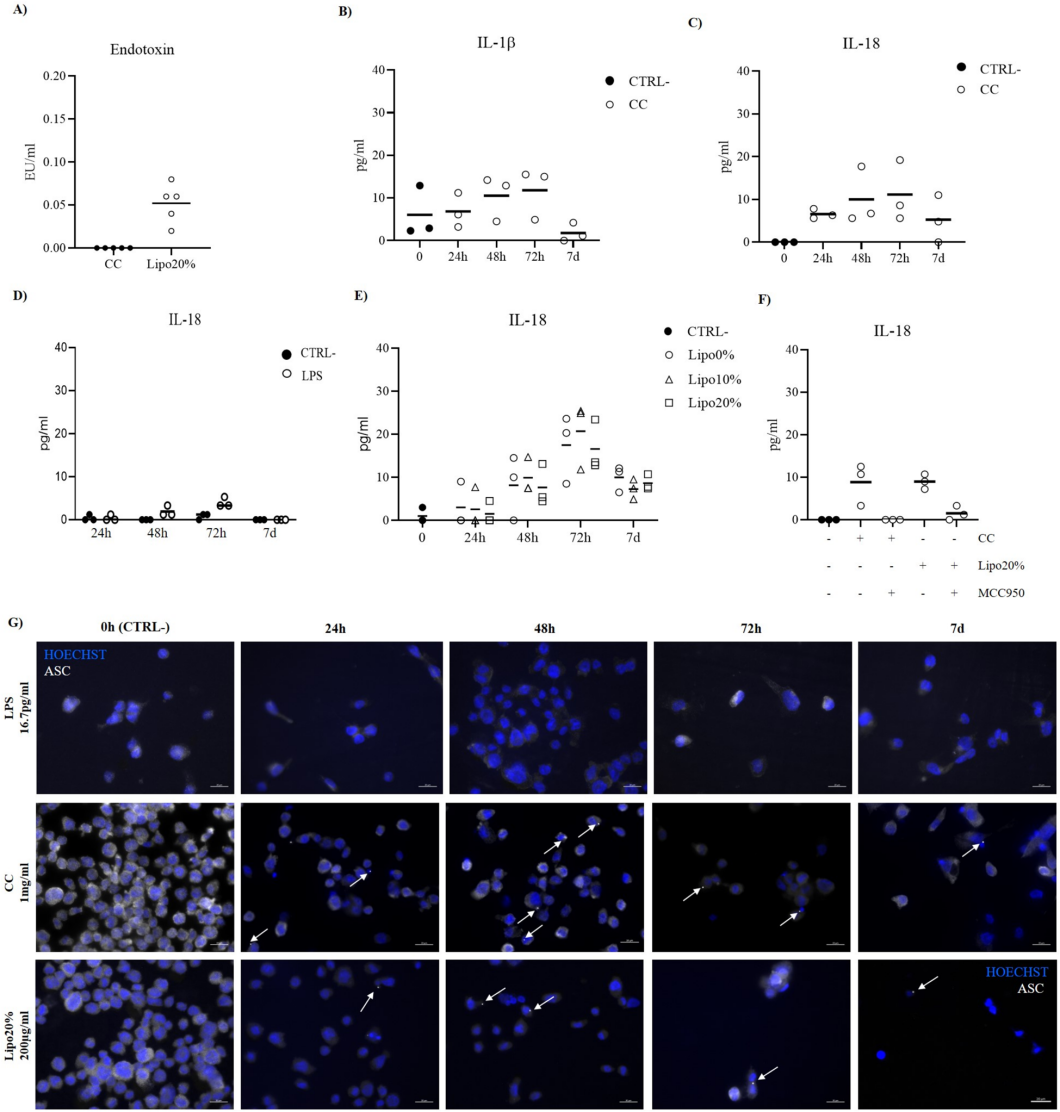
Cholesterol-mediated NLRP3 inflammasome activation in MLC. **A** Detection of endotoxin levels in cholesterol crystals (CC) 1 mg/ml and liposomes 200 µg/ml at 20% of cholesterol (Lipo20%); data shown as mean of *N* = 5 independently prepared stimuli; EU, endotoxin units. ELISA analysis of **B** IL-1β and **C** IL-18 in supernatant of MLC incubated with CC 1 mg/ml for t 0 h (CTRL −), 24 h, 48 h, 72 h, or 7 days (7 d); **D** IL-18 in supernatant of MLC untreated (CTRL −) or treated with LPS 16.7 µg/ml (LPS) with a final endotoxin concentration of 0.05 EU/ml, to reproduce the endotoxin contamination level in liposomes; **E** IL-18 in supernatant of MLC treated with liposomes 200 µg/ml at 0% (Lipo0%), 10% (Lipo10%), or 20% (Lipo20%) of cholesterol; **F** IL-18 in supernatant of MLC treated with CC or Lipo20% for 72 h alone or after preincubation with NLRP3 inflammasome inhibitor MCC950 at 10 µM. For **B**, **C**, and **E**, data are reported as mean of three independent experiments in different plates with three different cell passages and freshly prepared stimuli (*N* = 3). For **D** and **F**, data are shown as mean of three technical replicates. **G** Immunofluorescence analysis of ASC specks. Representative results of ASC speck formation in MLC treated with LPS 16.7 µg/ml (LPS) with a final endotoxin concentration of 0.05 EU/ml (upper panels), with CC 1 mg/ml (middle panels) or with cholesterol-enriched liposomes 200 µg/ml at 20% cholesterol (Lipo20%, lower panels) for 0 h (CTRL −), 24 h, 48 h, 72 h, or 7 d. Cells were fixed, then stained with anti-ASC antibody, followed by staining with PE-conjugated secondary antibody (false colored in white). ASC specks are indicated by white arrows. Nuclei were stained with Hoechst 34,580 (blue). All the micrographs were taken at the same magnification and reported with the same scale (scale bar = 20 µm). Individual data values are provided in an additional excel file (Additional file 2: Supporting data values)

To estimate if the uptake of cholesterol in CC form or in cholesterol-enriched liposomes triggers the NLRP3 inflammasome activation and release of inflammatory cytokines in unprimed MLC, the production of pro-IL-1β, the extracellular release of cytokines in cell supernatant (IL-1β and IL-18), and the formation of ASC speck were detected at different time points. To exclude biases in inflammasome activation due to contamination of the applied stimuli, an analysis of the endotoxin level was performed. No endotoxin was detected in CC samples, while a mean level of endotoxin of 0.05 EU/ml (± 0.02) was found in the liposome samples (Fig. 3A). To recapitulate the possible effect of the endotoxin, MLC were treated with LPS at 0.05 EU/ml (corresponding to 16.7 pg/ml LPS) for the same time exposure used for the liposome experiments. The levels of IL-18 and IL-1β were detected by ELISA and reported in Fig. 3D and in supplementary material (Additional file 1: Fig. S3A), respectively, and the immunodetection of ASC speck was performed. Since the effect of the endotoxin at this concentration on the level of IL-1β cannot be considered negligible compared to the effect of the liposomes (Additional file 1: Fig. S3B), IL-1β was not be used as a readout for the inflammasome activation in liposome experiments. On the other hand, not appreciable effect of the endotoxin at 0.05 EU/ml was found on the release of IL-18 (Fig. 3D), and no ASC speck was detected at any time points (Fig. 3G, upper panels).

Although we were unable to detect pro-IL1β intracellularly in any of the treatment groups (Additional file 1: Fig. S3C), probably due to the sensitivity of the WB method, a time-dependent release of both IL-1β and IL-18 was detected in the supernatant of MLC treated with CC, with the highest levels reached at 72 h (Fig. 3B). Of note, while a basal level of IL-1β can be detected at the time 0 h in the MLC model, due to residual effect of PMA treatment [29], no basal levels of IL-18 are detectable in the in vitro model, pointing to the CC treatment as the sole activating agent for the release of IL-18. The activation status of the NLRP3 inflammasome was confirmed by detection of ASC speck by immunofluorescence (Fig. 3G). MLC treated with LPS at 5 µg/ml served as technical positive control for the validation of the method (Additional file 1: Fig. S4A, CTRL +). The ASC protein is constitutively expressed in macrophages and distributed in the cytoplasm. When the NLRP3 inflammasome is activated, the ASC forms a complex with NLRP3 and pro-caspase-1, known as ASC speck, detected as a perinuclear 1-µm dot. At 0 h, a diffuse signal for ASC was present throughout the cell and no ASC speck was detected (Fig. 3D, 0 h (CTRL −)). After treatment with CC, ASC speck was detected at all time points (Fig. 3D, middle panels), confirming that the release of cytokines was inflammasome-dependent. CC were previously reported to promote inflammatory responses including NLRP3 inflammasome activation with subsequent release of pro-inflammatory cytokines in bone marrow-derived macrophages primed with Pam3csk4 [36], LPS-primed monocyte-derived macrophages, and THP-1-derived macrophages [28]. However, in previous studies, the release of IL-1β was LPS-dependent or sustained by PMA. To exclude biases in NLRP3 activation due to the priming agent, here we tested the hypothesis of a CC-mediated inflammasome activation in unprimed MLC. Thus, the activation of the inflammasome in our study is merely CC-dependent.

For experiments with liposomes, liposomes without cholesterol (Lipo0%) or enriched with physiological (Lipo10%) or supraphysiological (Lipo20%) content of cholesterol were used. A time-dependent release of IL-18 was found, and ASC speck was detected from t24h in treatment groups (Fig. 3D lower panels and Additional file 1: Fig. S4B). However, no difference was found among liposomes at different concentrations of cholesterol, indicating an effect of the lipids rather than a specific response to cholesterol. Indeed, upregulated production of phosphatidylcholine was previously associated to NLRP3 inflammasome activation and IL-1β and IL-18 production in macrophages [37].

To further investigate the role of NLRP3 inflammasome, MLC were treated with CC or Lipo20% for 72 h alone or after preincubation with NLRP3 inflammasome inhibitor MCC950. ELISA analysis of IL-18 confirmed that the level of cytokine decreases in the presence of the inhibitor (Fig. 3F).

For both CC and cholesterol-enriched liposome experiments, no gasdermin D (GSDMD), activator of pyroptosis, was detected at any time points (Additional file 1: Fig. S3C). Accordingly, the viability was higher than 90% in all treatment groups (Additional file 1: Fig. S3D).

These data confirm that CC and lipid overload cause activation of the inflammasome and NLRP3-dependent release of IL-18 in unprimed MLC.

MLC experiments reported here were analyzed using descriptive statistics since these experiments do not serve the purpose of testing a hypothesis but rather optimizing the experimental conditions to run the final experiments in cells isolated from animals. While MLC experiments contribute with biological relevance to this work, there are many limitations on the use of a cell line, which is intrinsically homogenous, even at different passages, and the definition of an independent experimental unit in cell culture [38]. In the following experiments, every analyzed cell sample comes from an independent animal subject, complying with the definition of an independent biological unit or replicate, and the statistical power was ensured by previously running a sample size analysis. Therefore, conclusions are related to the last two sections, performed with isolated cells from animals, contributing to the biological and physiological relevance of this work.

### Uptake of cholesterol causes foam cell formation in primary AM

Altogether, the results obtained in the in vitro experiments in MLC point towards 72 h as the time point in which treatment with CC and Lipo20% causes changes in cholesterol metabolism, foam cell formation, activation of the NLRP3 inflammasome, and inflammasome-dependent release of IL-18. To explore whether the same stimuli cause a response also in AM, the treatments were applied for 72 h directly in mouse primary AM.

**Fig. 4:**
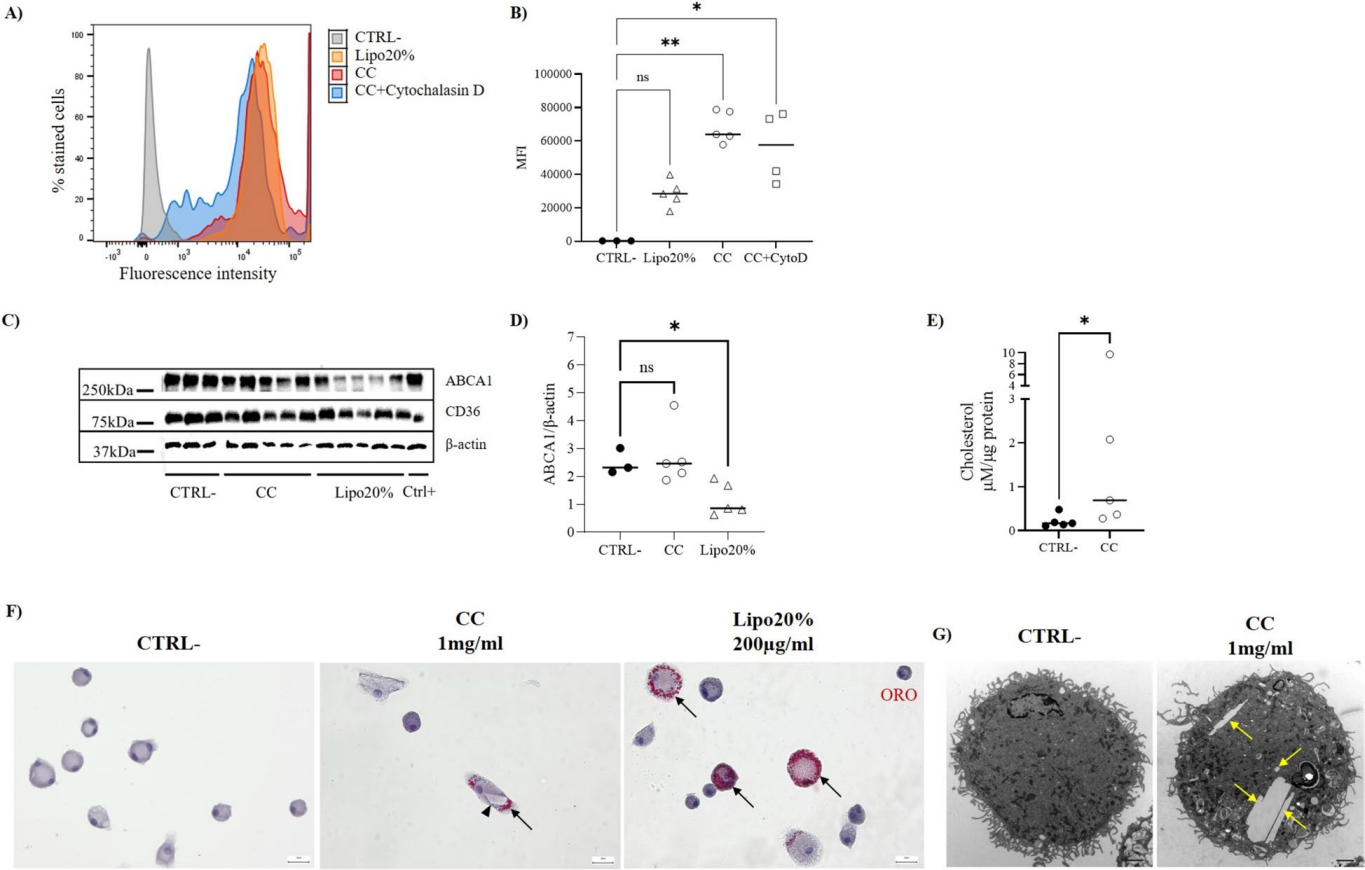
Cholesterol uptake, accumulation, and metabolism in primary AM. **A**, **B** FACS analysis of AM untreated (CTRL −, gray) or treated for 24 h with bodipy-labeled cholesterol-enriched liposomes 200 µg/ml at 20% cholesterol (Lipo20%, orange), bodipy-labeled CC at 1 mg/ml alone (CC, red), or with cytochalasin D (CC + cytochalasin D, blue) at 5 µM 10 min prior and during treatment with 1 mg/ml CC. **A** Representative histogram plots of y: percentage of stained cells normalized by mode vs x: fluorescence intensity; **B** mean fluorescence intensity (MFI) for the different treatment groups. CytD, cytochalasin D. **C** Western blot analysis for proteins of the RCT pathway and β-actin and **D** densitometry analysis of ABCA1 to the loading control (β-actin) in AM cell lysate untreated (CTRL −) or incubated with CC 1 mg/ml or Lipo20% for 72 h. **E** Fluorometric cholesterol quantification of AM untreated (CTRL −) or treated with CC at 1 mg/ml for 72 h. **F** Oil Red O (ORO) staining of AM untreated (CTRL −) or treated with CC 1 mg/ml (CC) or with cholesterol-enriched liposomes 200 µg/ml at 20% cholesterol (Lipo20%) for 72 h. Intracellular neutral lipid droplets are stained in red, cells are counterstained with Mayer’s hematoxylin (blue), and foam cells are indicated by black arrows. In the CC group, a crystal (arrowhead) can be observed in the cytoplasm of an AM along with ORO-stained lipid droplets. Scale bar 20 μm. **G** Representative TEM micrographs of MLC untreated (CTRL −) or treated with CC 1 mg/ml for 72 h. Internalized CC are indicated by yellow arrows. All the micrographs are reported with the same scale (scale bar 1 μm). Statistical analysis: **B**–**D** Kruskal–Wallis test followed by Dunn’s multiple comparisons, **E** Mann–Whitney test; *p < 0.05, **p < 0.01. Data shown as median and plot of the individual values (*N* = 3–5). Individual data values are provided in an additional excel file (Additional file 2: Supporting data values)

The ability of AM to uptake cholesterol in the form of CC or cholesterol-enriched liposomes was evaluated by FACS after 24-h exposure to the stimuli. Based on the results reported above, AM were preincubated with cytochalasin D, as potential inhibitor for CC uptake. After incubation, treated AM were 100% fluorescence-positive, and the increase in MFI was statistically significant for the CC group compared to control group (*p* = 0.004). Moreover, the uptake was dose-dependent as shown by a lower MFI in AM treated with Lipo20% (containing a total amount of cholesterol of 40 µg/ml), compared to CC (1 mg/ml cholesterol) (Fig. 4A and B). Furthermore, a decrease in MFI was observed in the cytochalasin D group compared to the CC group, confirming that also for AM, phagocytosis plays a role in the uptake of CC. Of note, for AM treated with CC alone or with CC after pretreatment with cytochalasin D, 13% of total events had off-the-chart intensity that could have affected the accuracy of the reported MFI values. On the other hand, no changes were found in CD36 level after 72-h exposure (Fig. 4C), excluding a role of this receptor in the uptake of CC, also after longer exposure. Interestingly, a similar result was obtained for silica-induced lung inflammation in mice, in which inhibition of CD36 in mice did not reduce silica-induced IL-1β production in bronchoalveolar lavage (BAL) fluid [39], consistent with the ability of AM from CD36-deficient mice to normally recognize silica crystals [40]. It has been proposed that this may be due to the marginal CD36 expression on resident alveolar macrophages [39], while the receptor may contribute to the chronic lung inflammation [22], where recruited monocyte-derived macrophages with higher expression of CD36 infiltrate alveolar spaces [41].

Unfortunately, we were unable to detect ApoE by western blot in the AM in any of the treatment groups, nor in the technical positive control used for the validation of the mouse antibodies (Ctrl +, AM treated with 25 µg/ml oxLDL). Therefore, we cannot comment on the role of ApoE in AM.

For ABCA1, no difference was found between CC group and untreated AM (CTRL −), while a statistically significant decrease (*p* = 0.046) was found for AM treated with Lipo20% (Fig. 4D). ABCA1 is a transporter protein previously described in the lung in AM and AT2C [42] and involved in receptor-mediated cholesterol efflux cell membrane, by mediating the release of phospholipid and cholesterol to lipid-free apolipoproteins, thus maintaining cellular cholesterol homeostasis. In our study, the lower levels of the protein in Lipo20% AM may be due to the nature of the liposomes. Indeed, long-chain unsaturated fatty acids, such as the 18:1 chain of the 1-palmitoyl-2-oleoyl-sn-glycero-3-phosphoglycerol (POPG) in our liposomes, decrease ABCA1 expression in murine macrophages by increasing its protein degradation rate [43]. Moreover, it is significantly downregulated in lungs of patients with moderate to severe COPD [44], in which also foam cells were previously described [45].

On the other hand, AM exposed to CC and Lipo20% showed foam cell formation (Fig. 4F), a characteristic feature recently described in lungs of patients with a variety of pulmonary diseases, such as interstitial lung diseases [46], tuberculosis [47], and chronic obstructive pulmonary disease [45]. Moreover, AM exposed to CC showed intracellular increase of cholesterol (Fig. 4E, p = 0.032) and cytoplasmic lipid droplets and crystal accumulation, as confirmed with both ORO staining and TEM (Fig. 4F and G). Of note, our group recently reported the case of a patient affected by IPF in which electron microscopy of the lung demonstrated the presence of intraparenchymal macrophages loaded with crystals [16]. Although the nature of the crystals is unknown, they resemble in shape to the cholesterol cleft normally reported in atherosclerosis.

Taken together, these data confirm the ability of AM to uptake soluble cholesterol and CC, the latter presumably through phagocytosis, leading to foam cell formation after sustained exposure. However, at the chosen cholesterol concentration, no activation of the RCT pathway was detected.

### CC activate the NLRP3 inflammasome in AM

**Fig. 5:**
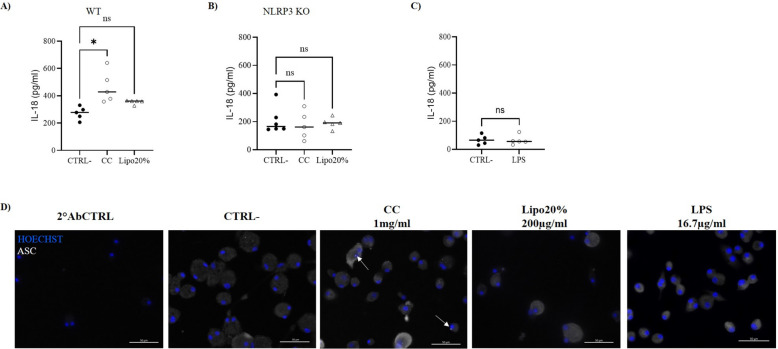
Cholesterol-mediated NLRP3 activation in primary AM. ELISA analysis of IL-18 in supernatant of AM isolated from **A** wild type (WT) or from **B** NLRP3 knockout (KO) mice and left untreated (CTRL −) or incubated with CC 1 mg/ml or with liposomes 200 µg/ml at 20% of cholesterol (Lipo20%) for 72 h. **B** ELISA analysis of IL-18 in supernatant of AM isolated from NLRP3 knockout (KO) and left untreated (CTRL −) or incubated with cholesterol crystals (CC) 1 mg/ml or with liposomes 200 µg/ml at 20% of cholesterol (Lipo20%) for 72 h. Statistical analysis: Kruskal–Wallis test followed by Dunn’s multiple comparisons, *p < 0.05, ns: non-significant. **C** ELISA analysis of IL-18 in supernatant of WT AM untreated (CTRL −) or treated with LPS 16.7 µg/ml with a final endotoxin concentration of 0.05 EU/ml (LPS), to reproduce the endotoxin contamination level in liposomes. Statistical analysis: Mann–Whitney test, ns: non-significant. **A**–**C** Data shown as median and plot of the individual values (*N* = 5). **D** Immunofluorescence analysis of ASC specks. Representative results of ASC speck formation in AM untreated (CTRL −) or treated with CC 1 mg/ml (CC), or with cholesterol-enriched liposomes 200 µg/ml at 20% cholesterol (Lipo20%), or treated with LPS 16.7 µg/ml with a final endotoxin concentration of 0.05 EU/ml (LPS) for 72 h. Cells were fixed and stained with anti-ASC antibody, followed by staining with PE-conjugated secondary antibody (false colored in white). ASC specks are indicated by white arrows. Nuclei were stained with Hoechst 34,580 (blue). As a negative control, cells were incubated with the secondary antibodies alone (2ndAbCTRL). All the micrographs were taken at the same magnification and reported with the same scale (scale bar = 20 µm). Individual data values are provided in an additional excel file (Additional file 2: Supporting data values)

Cholesterol-mediated activation of the inflammasome has been extensively investigated in the atherosclerosis field, while still little is known about this mechanism in the lung. Here, we investigated whether soluble cholesterol and/or CC trigger the sterile production of pro-inflammatory markers in AM. As shown in Fig. 5A and B, respectively, a statistically significant increase (*p* = 0.003) of IL-18 was found in supernatant of AM exposed to CC and a slight increase was found in AM treated with Lipo20%. To reproduce the possible effect of the endotoxin contamination in the liposomes, AM were left untreated (CTRL −) or treated with LPS (LPS) at 0.05 EU/ml (corresponding to 16.7 pg/ml LPS) in serum-free medium for 72 h. The levels of IL-18 were detected in supernatant by ELISA and the presence of ASC speck was investigated. No appreciable effect of the endotoxin at this concentration was found on the release of IL-18 (Fig. 5C), and no ASC speck was detected (Fig. 5D, LPS).

No IL-1β was detected instead, not in AM treated with CC nor with Lipo20%, nor in the LPS-treated AM, serving as technical positive control. Of note, although the two cytokines share several biological properties, they differ in the signaling pathway. Indeed, high concentrations of IL-18, in the order of nanomolar, are required to activate a response, versus much lower concentrations of IL-1β that is active in the low picomolar range [48, 49]. Moreover, IL-1β was reported to have a short half-life in blood [50]. The use of commercially available ELISA kit and long exposure to the stimuli may limit the detection of IL-1β.

To estimate if the IL-18 is NLRP3-dependent, the same stimuli were applied to AM isolated from NLRP3 knockout mice. In this case, none of the stimuli had effect on the release of IL-18 (Fig. 5B) and the levels of the cytokine were comparable among the three groups. Moreover, the activation of NLRP3 inflammasome following exposure to CC and Lipo20% was investigated by immunofluorescence detection of ASC speck. CC, but no Lipo20%, stimulated the assembly of the inflammasome in speck (Fig. 5D), suggesting that the crystalline structure is needed for the sterile activation of the inflammasome in unprimed AM. Indeed, crystals and particulate matter, such as monosodium urate, silica, and calcium crystals, induce NLRP3 inflammasome activation in macrophages, through damage of lysosome and release of lysosome content into the cytoplasm after phagocytosis, as reviewed by Kelley et al. [51]. Remarkably, the applied stimulus was made of chemically pure endotoxin-free synthetic CC, providing strong proof that CC themselves were the solely active agent for the triggering and the activation of the inflammasome. It was already demonstrated that crystalline cholesterol acts as an endogenous danger signal activating the NLRP3 and thus contributing to atherogenesis [23]. Here, we reported that AM are able to initiate an analogous mechanism, opening a new scenario for the possible role of AM and of cholesterol-mediated inflammation in the lung.

## Conclusions

In this study, the role of cholesterol, in soluble or crystalline forms, as activating agent for NLRP3 inflammasome in AM was investigated. By performing in vitro and ex vivo investigations, including AM from NLRP3 knockout animals, we here provide robust evidence that cholesterol crystals, but not soluble cholesterol, are able to trigger the assembly and activation of the inflammasome, thus leading to the inflammasome-dependent release of IL-18. To the best of our knowledge, this is the first time that this mechanism is proven directly in AM.

## Methods

### Cholesterol crystal preparation

Cholesterol powder was dissolved in warm acetone (12.5 mg/ml) and then cooled down overnight at room temperature (RT) to precipitate cholesterol [28]. After 4 cycles of recrystallization, the crystals (size range of 1–5 mm) were collected, ground using a sterile mortar and a pestle to yield a size range of 1–10 µm. The final size was assessed by SEM, as described below. Obtained crystals will be autoclaved for 15 min at 121 °C, then stored at − 20 °C until use.

Endotoxin was detected in the crystals by Limulus Amebocyte Lysate QCL-1000 assay to exclude triggering of the inflammation activation due to contamination of reagents.

Fluorescence-labeled CC were obtained by addition of BODIPY-conjugated cholesterol (Cayman, MN, USA) at a final concentration of 1% of total cholesterol.

### Liposome preparation

All the lipids were purchased from Avanti Polar Lipids (AL, USA). Liposome suspensions was produced by mixing the main phospholipid species of surfactant lipids, 1,2-dipalmitoyl-sn-glycero-3-phosphocholine (DPPC) and POPG, at a proportion of 7:3 (DPPC/POPG, w/w), widely used as a model system because it mimics roughly the lipid composition of lung surfactant [52]. Liposomes were produced without cholesterol (0 wt.%, Lipo0%) or supplemented with physiological (~ 10 wt.%, Lipo10%) or supraphysiological amounts of cholesterol (~ 20 wt.%, Lipo20%) [16, 53]. Lipids were mixed in chloroform–methanol (2:1; v/v), then dried out under a nitrogen stream. Multilamellar suspensions were prepared by rehydrating samples in Tris buffer (5 mM Tris, 150 mM NaCl, pH 7) at 50 °C for 1 h with intermittent shacking [54].

Endotoxin was detected in liposomes by Limulus Amebocyte Lysate QCL-1000 assay to exclude triggering of the inflammation activation due to contamination of reagents.

Fluorescently labeled cholesterol-enriched liposomes were obtained by addition of BODIPY-conjugated cholesterol (Cayman, MN, USA) at a final concentration of 1% of total cholesterol.

### Cell culture

THP-1 (ATCC® TIB202™, Virginia) monocytes were cultured in RPMI-1640 complete medium with L-glutamine, 25 mM HEPES (Corning, Virginia) containing 10% fetal bovine serum (FBS, Sigma Aldrich, Germany) and 1% penicillin/streptomycin (10,000 units penicillin + 10 mg streptomycin/ml, Sigma Aldrich, Germany), plated at a density of 2 × 10^5^ cells/ml in T25 flasks (Eppendorf, Germany), and incubated at 37 °C in 5% CO_2_.

THP-1 monocytes were induced to differentiate to MLC by incubation of 5 × 10^5^ cells in a 6-well plate in 2 ml complete medium with 5 ng/ml of PMA (Stemcell technology, Switzerland) in dimethyl sulfoxide (final concentration 0.1%, DMSO, Sigma Aldrich, Germany) for 48 h plus 24 h in fresh medium, as previously described [29]. MLC, differentiated following the method here reported, were previously validated as a valuable method to study the inflammasome [29], and allowed us to properly select the conditions to be replicated in the murine AM, in order to reduce the number of animals involved in the study.

### Experimental and control treatments

For the treatment, MLC were cultured at 37 °C under 5% CO_2_ in a serum-free culture medium in presence of the following stimuli: preformed CCs at 1 mg/ml [23, 28, 55] for 0 h, 24 h, 48 h, or 7 days; liposomes at cholesterol concentration of 0 wt.% (Lipo0%), 10 wt.% (Lipo10%), and 20 wt.% (Lipo20%) (final total lipid concentration of 200 µg/ml [56], 20 μg/10^5^ cells) for 0 h, 24 h, 48 h, or 7 days. For experiments of 7-day duration, the medium was replaced after 72 h and a fresh stimulus will be applied. Control treatments included: MLC incubated at 37 °C, 5% CO_2_ in the presence of fluorescently labeled oxLDL (oxLDL-DyLight 488, 1:50 dilution, Cayman, MN, USA) for 24 h alone as technical positive control, or with the CD36 inhibitor myricetin (Cayman, MN, USA) 25 µM 2 h prior oxLDL, as technical inhibitor control for cholesterol uptake analysis; MLC treated with the phagocytosis inhibitor cytochalasin D (RayBiotech, GA, USA) at 5 µM 10 min before [31], or CR3 inhibitor compstatin (Selleckchem, Germany) at 20 µM 5 min before [33] and during treatment with CC, or with cytochalasin D and myricetin before and during treatment with cholesterol-enriched liposomes as inhibitor groups in the uptake assay; MLC incubated at 37 °C, 5% CO_2_ in the presence of 25 or 50 µg/ml oxLDL for 24 h as technical control for the RCT pathway and for foam cell formation [57], respectively; MLC treated with LPS (MLC + LPS) from *Escherichia coli* O26: B6 ≥ 10,000 EU/mg (Sigma Aldrich, Germany) (5 µg/ml, 15*10^3^ EU/ml, in phosphate-buffered saline, PBS) for 3 h in complete media as technical positive control of NLRP3 inflammasome activation through ASC speck formation and IL-1β production, as previously described [23]; MLC incubated with the NLRP3 inhibitor MCC950 (Sigma Aldrich, Germany) at 10 µM in DMSO [58] or vehicle (0.1% DMSO) 30 min before and during treatment with CC and cholesterol-enriched liposomes as inhibition group to NLRP3 activation.

### Primary alveolar macrophages

Wild type (strain C57BL/6J) and NLRP3 knockout (KO, background C57BL/6J) mice are inbred and kept in the animal facilities of the Charité Universitätsmedizin Berlin. Primary AM were isolated from wild type and from NLRP3 knockout mice and cultivated as previously described [16, 59]. Briefly, AM were harvested by BAL, after animal sacrificing, procedure approved by institutional authorities (“Tierschutzbeauftragte” and “Tierschutzausschuss” of the Charité—Universitätsmedizin Berlin, Germany) with the permission of local governmental authorities (State Office for Health and Social Affairs Berlin, Germany). BAL was obtained by cannulation of the trachea, followed by a thoracotomy. The pulmonary vasculature was rinsed via the right ventricle with 0.25% heparin in 0.9% NaCl. Subsequently, three 1.5 ml aliquots of pre-warmed NaCl with 2 mM EDTA and 0.5% FBS (BAL buffer) were actively instilled until the lungs were fully inflated. The liquid was withdrawn and collected in 15-ml tubes containing RPMI 1640 complete medium. Cells were collected by centrifugation at 300 × g 5 min at 4 °C, resuspended in 500 μl BAL buffer, counted, plated at 3–4 × 10^5^ cells per well of 6-well plate, or at 2 × 10^5^ cells per well of 12-well plate, or at 1 × 10^5^ cells per well of a 24-well plate in 3 ml, 1.5 ml, or 1 ml pre-warmed complete culture medium, respectively, supplemented with primocin (InvivoGen, CA, USA), and incubated at 37 °C, 5% CO_2_ overnight. Medium was then replaced with RPMI 1640 complete medium with 5 ng/ml purified recombinant mouse GM-CSF and cells were let grow until confluence (medium change every 2 days). Typically, total of ~ 3–5 × 10^5^ AM cells can be obtained per adult mouse [60].

To validate the results on MLC, primary AM at confluence were treated with one of the conditions tested in MLC (CC or cholesterol-rich liposomes at 10 wt.% or 20 wt.% cholesterol) and the same time points reported for MLC. The condition activating NLRP3 inflammasome in MLC study was chosen as treatment for the AM and the samples were analyzed using the same methods and readout parameters.

### Scanning electron microscopy

The CC samples were prepared for SEM by air drying. Since previous studies demonstrated rapid dissolution of crystals with solvents, standard ethanol series dehydration and critical point drying were omitted [61]. CC were attached to pin stubs with double-sided conductive tape and sputtered using a sputter coater (MED-020 high-vacuum coating system, Baltec, Germany) with gold palladium. The samples were examined using a scanning electron microscope (GeminiSEM300, Zeiss, Germany) with a secondary electron detector at 7 kV.

### Transmission electron microscopy

Intracellular presence of CC was assessed by transmission electron microscopy (TEM). Before and after treatment with CC and cholesterol-enriched liposomes, MLC were detached by trypsinization, collected by centrifugation (300 × g, for 5 min), and the cell pellet was washed in PBS and fixed with 1.5% paraformaldehyde and 1.5% glutaraldehyde (Serva, Germany) in 0.15 M Hepes buffer. After washing in 0.1 M cacodylate buffer, the samples were post-fixed in 1% OsO4 (Electron Microscopy Sciences, USA) in 0.1 M cacodylate buffer at RT for 1.5 h and washed again twice in cacodylate buffer. The samples were then included in agarose overnight at 4 °C, followed by incubation in 4% aqueous uranyl acetate (Merck, Burlington, USA) overnight at RT. After dehydration in a graded acetone series, the samples were embedded in EPON resin (Roth, Germany). Finally, sliced ultrathin sections of 70 nm thickness were stained with uranyl acetate and lead citrate. Samples were examined using Zeiss EM 906 at 80 kV acceleration voltage (Zeiss, Germany).

### Protein concentration analysis

At collection time, cell pellets were lysed by addition of RIPA lysis buffer (Thermo Scientific, Germany) containing protease inhibitor cocktail (cOmplete, Sigma Aldrich, Germany) in constant agitation for 30 min at 4 °C. Cell membranes were separated by centrifugation at 12,000 × g for 20 min at 4 °C.

The protein concentration in cell lysates and cell membranes were determined using the BCA™ Protein Assay Kit (Thermo Fisher Scientific, Germany).

### Intracellular cholesterol content quantification

Intracellular content of total cholesterol was measured by commercially available fluorometric assay (Total Cholesterol Assay Kit, STA-390, Cell Biolabs Inc., CA, USA). Briefly, cell extract was obtained with chloroform:isopropanol:NP-40 (7:11:0.1) in a micro-homogenizer. After centrifugation (15,000 × g, 10 min), the organic phase was transferred in a new tube and dried out at 50 °C under nitrogen stream. The samples were then resuspended in assay diluent and total cholesterol concentration was quantified, following manufacturer’s instructions. Fluorescence was read with a fluorometric plate-reader (Fluostar Omega, BMG Biotech, Germany). Data were normalized by total protein concentration in cell lysate.

### Uptake of CC and cholesterol-enriched liposomes

The ability of MLC to uptake cholesterol in form of CC or cholesterol-enriched liposomes was evaluated by fluorescence-activated cell sorting analysis (FACS). MLC were treated with fluorescence-labeled CC or fluorescence-labeled cholesterol-enriched liposomes for 12 h or 24 h, with or without uptake inhibitors. At the time of collection, MLC were detached by trypsinization, collected by centrifugation (300 × g, for 5 min), and the cell pellet was washed twice in 5% FBS in PBS. The percentage of fluorescence-positive cells were evaluated by a BD FACS Canto™ II Cell Analyzer (BD Biosciences, USA). To distinguish between internalized and surface-bound cholesterol, the cells were quenched with trypan blue (1.2 mg/ml) shortly before flow cytometry analysis [62]. For every sample, 20,000 events will be recorded. Cells were gated by forward scatter—area (FSC-A) vs side scatter—area (SSC-A), then by SSC-A vs side scatter—width (SSC-W) and by FSC-A vs forward scatter—width (FSC-W). Flow cytometric data were analyzed by FlowJo™ software (v10.7.2, BD, OR, USA).

### Foam cell formation

Foam cell formation were evaluated by ORO staining, as previously described [57]. Briefly, the ORO stock solution was prepared by dissolving 0.5 g ORO powder/100 ml in warm (56 °C) isopropyl alcohol. Immediately before use, the stock solution was pre-warmed at 60 °C, filtered by filter paper (Whatman, Germany), and diluted 3:2 (v/v) with distilled water (ORO working solution). MLC were seeded at a density of 1.5 × 10^5^ cells/ml in a 24-well plate containing a glass coverslip per well and treated accordingly. At the collection time, the cells were fixed for 10 min in 10% neutral buffered formalin, gently rinsed once with PBS followed by 60% isopropyl alcohol (15 s), and stained with ORO working solution at 37 °C for 1 min in the darkness. The excess of staining was removed by quick rinse in 60% isopropyl alcohol (15 s), followed by three washes in PBS. The cells were counterstained with Mayer’s hematoxylin for 1 min, to stain the nuclei, followed by bluing in tap water [63]. The coverslips were mounted on a microscope slide using an aqueous-based mounting medium. Images will be acquired by Axio Imager.Z1 (Zeiss, Germany) and analyzed by ZEN software (v3.4, blue edition, Zeiss, Germany).

### Western blot

Cell pellet was lysed as reported above. Proteins were denatured in 1 × Laemmli buffer (Tris–HCl 63 mM, glycerol 10%, SDS 2%, bromophenol blue 0.01%, 2-mercaptoethanol 5%; pH 6.8) by heating for 10 min at 95 °C, then resolved by SDS-PAGE, with 8, 12, or 16% (wt/vol) separating gels, and transferred to PVDF membranes. Nonspecific sites on PVDF membrane were blocked with 5% non-fat milk powder (VWR, Belgium), or 5% bovine serum albumin in TBS + 0.1% Tween for 1 h at RT. The membranes were incubated overnight at 4 °C with primary antibody diluted in blocking solution. Proteins were detected by incubation with HRP-conjugated secondary antibodies at half of the primary antibody concentration, and protein bands visualized by ECL western blotting detection reagents (Amersham Pharmacia Biotech, UK) and enhanced chemiluminescence (ECL Chemocam imager, Intas science imaging, Germany). β-Actin served as loading control and was analyzed following the same protocol.

To detect changes in RCT pathway following treatments, levels of CD36, liver X receptor (LXR), SREBP-2, ApoE, and ABCA1 were detected in cell lysate. MLC treated with oxLDL at 25 µg/ml served as technical positive control.

The effect of the treatments on the NLRP3 inflammasome was evaluated through changes in pro-IL-1β levels in cell lysate and cleaved GSDMD levels in cell membranes.

The list of antibodies used in the study is reported in supplementary material (Additional file 3: Tables S1 and S2.).

### ELISA

Commercially available ELISA kits were used to measure the levels of secreted IL‐1β (Human IL-1beta/IL-1F2, DY201; Mouse IL-1beta/IL-1F2, DY401-05, R&D System, MN, USA) and IL-18 (Human Total IL-18, DY318-05; Mouse IL-18, DY7625-05, R&D System, MN, USA) in undiluted cell media, according to the manufacturer’s instructions.

### Immunohistochemistry of ASC speck

ASC speck oligomerization was detected by immunofluorescence as a readout of inflammasome activation, as previously described [29, 64]. Briefly, MLC were seeded at a density of 1.5 × 10^5^ cells/ml in a 24-well plate containing a glass coverslip per well and treated accordingly. At the collection time, cells adherent to the coverslip were washed in PBS and fixed in PBS-buffered formalin 4% for 30 min at 37 °C, then blocked in blocking/permeabilization (block/perm) buffer (10% goat serum, 1% FBS, and 0.5% Triton- × 100 in PBS) for 30 min at 37 °C. The cells were then incubated with anti-ASC/TMS1 primary antibody in block/perm buffer for 1 h at RT, followed by incubation with conjugated secondary antibody in block/perm buffer for 1 h at RT. Nuclei were stained by incubation with Hoechst 34580 (Chemodex, Switzerland) in PBS for 30 min at RT. Images were acquired by Axio 196 Imager.Z2 (Zeiss, Germany) and analyzed by ZEN software (v3.4, blue edition, Zeiss, Germany). Signal due to secondary antibody unspecific binding was excluded by immunofluorescence analysis of the corresponding positive control (MLC + LPS) incubated with the secondary antibody alone in absence of primary antibody.

### Viability test

MLC were detached by trypsinization, collected by centrifugation (300 × g, for 5 min), and resuspended in 200 µl medium. An automated Corning Cell Counter (CytoSMART Technologies, Netherlands) was employed to calculate the number and viability of cells after trypan blue staining (1:1, v/v).

### Statistical analysis

For the experiments in MLC, every treatment condition was replicated in three different wells in the same row of the plate (technical replicates). The row represented the experimental unit. To obtain three biological replicates, the experiments were repeated three times in different plates with three different cell passages and freshly prepared stimuli (*N* = 3). For quantitative analyses, descriptive analysis was performed and data were reported as mean of the three biological replicates. For qualitative analyses, the experiments were performed in three technical replicates.

For experiments in primary AMs, required sample size analysis of animals per condition was performed by G*Power software (v 3.1.9.7) [65], based on the effect size calculated from the increase in cholesterol ester content in circulating macrophages after treatment with 1 mg/ml CC reported by Rajamäki and colleagues [28], minus 2 times standard deviation (2SD). The following protocol was applied for the calculation: *t*-test; effect size *d* = 2.7; *α* error probability = 0.05; power = 0.95. A minimum sample size per group of *N* = 4 is recommended. For more biological relevance for quantitative analyses, samples from 5 animals per group were analyzed. Non-parametric Kruskal–Wallis test followed by Dunn’s multiple comparisons test or Mann–Whitney test was performed to compare the treatment groups. Data are shown as median and the individual values were also plotted.

All the statistical analysis and graphs of the results were performed by GraphPad Prism 8 Software (San Diego, CA, USA).

## Supplementary Information

Additional file 1. Figures S1–S4. Fig. S1 Assessment of the cholesterol uptake by MLC. Fig. S2 Metabolism of cholesterol-enriched liposomes and foam cell formation in MLC. Fig. S3 NLRP3 inflammasome analysis and viability in MLC. Fig. S4 ASC speck detection

Additional file 2. Supporting data values: Figs. 1–5 and Figures S1–S3 (https://zenodo.org/records/17929307).

Additional file 3. Tables S1 and S2. Table S1 List of primary antibodies used for WB and/or immunofluorescence analysis. Table S2 List of secondary antibodies used for WB and/or immunofluorescence analysis

## Data Availability

All data generated or analyzed during the study are included in this published article and its supplementary information files. Individual data values are provided in an additional excel file (Additional file 2: Supporting data values) uploaded in Zenodo (with url: https://zenodo.org/records/17929307). In addition, all original blots included in this work can also be found in a file (called WB_Original blots_v2) uploaded also in Zenodo (with url: https://zenodo.org/records/17929307).

## Abbreviations

ABCA1: ATP-binding cassette transporter
AM: Alveolar macrophages
ApoE: Apolipoprotein E
AT2C: Alveolar epithelial type 2 cells
BAL: Bronchoalveolar lavage
CC: Cholesterol crystals
CR3: Complement receptor 3
DMSO: Dimethyl sulfoxide
DPPC: 1,2-Dipalmitoyl-sn-glycero-3-phosphocholine
*E. coli*: *Escherichia coli*
FACS: Fluorescence-activated cell sorting analysis
FBS: Fetal bovine serum
FSC-A: Forward scatter—area
FSC-W: Forward scatter—width
GSDMD: Gasdermin D
KO: Knockout
LDL: Low-density lipoprotein
Lipo0%: Liposomes without cholesterol
Lipo10%: Liposomes with 10% cholesterol
Lipo20%: Liposomes with 20% cholesterol
LXR: Liver X receptor
MFI: Mean fluorescence intensity
MLC: Macrophages-like cells
mol.%: Molar percent
NLRP3: NOD-like receptor protein-3
oxLDL: Oxidized low-density lipoprotein
PBS: Phosphate-buffered saline
PMA: Phorbol-12-myristate 13-acetate
POPG: 1-Palmitoyl-2-oleoyl-sn-glycero-3-phosphoglycerol
ORO: Oil Red O
RCT: Reverse cholesterol transport
RT: Room temperature
SEM: Scanning electron microscopy
Sftpc: Surfactant protein C
SRBP-2: Sterol regulatory element-binding protein- 2
SSC-A: Side scatter—area
SSC-W: Side scatter—width
wt.%: Weight percent
