# Supplementary material for: Cholesterol-mediated inflammation activation in alveolar macrophages
Source: BMC Biol. 2025 Dec 22;23:369. doi: 10.1186/s12915-025-02494-3 (PMC12750553; doi:10.1186/s12915-025-02494-3)
Supplement: Supplementary file 1 — Additional file 1. Figures S1–S4. Fig. S1 Assessment of the cholesterol uptake by MLC. Fig. S2 Metabolism of cholesterol-enriched liposomes and foam cell formation in MLC. Fig. S3 NLRP3 inflammasome analysis and viability in MLC. Fig. S4 ASC speck detection [file 12915_2025_2494_MOESM1_ESM.docx]

**Additional file 1**

**Cholesterol-mediated inflammation activation in alveolar macrophages**

Sonia Giambelluca^1,2^, Matthias Ochs^1,2^, Elena Lopez-Rodriguez^1,2*^

^1^ Institute of Functional Anatomy, Charité - Univeristätsmedizin Berlin, Berlin, Germany

^2^ German Center for Lung Research (DZL), Berlin, Germany

* Correspondence:

Elena Lopez-Rodriguez: [elena.lopez-rodriguez@charite.de](mailto:elena.lopez-rodriguez@charite.de)

**Supplementary figures**

**Fig. S1**


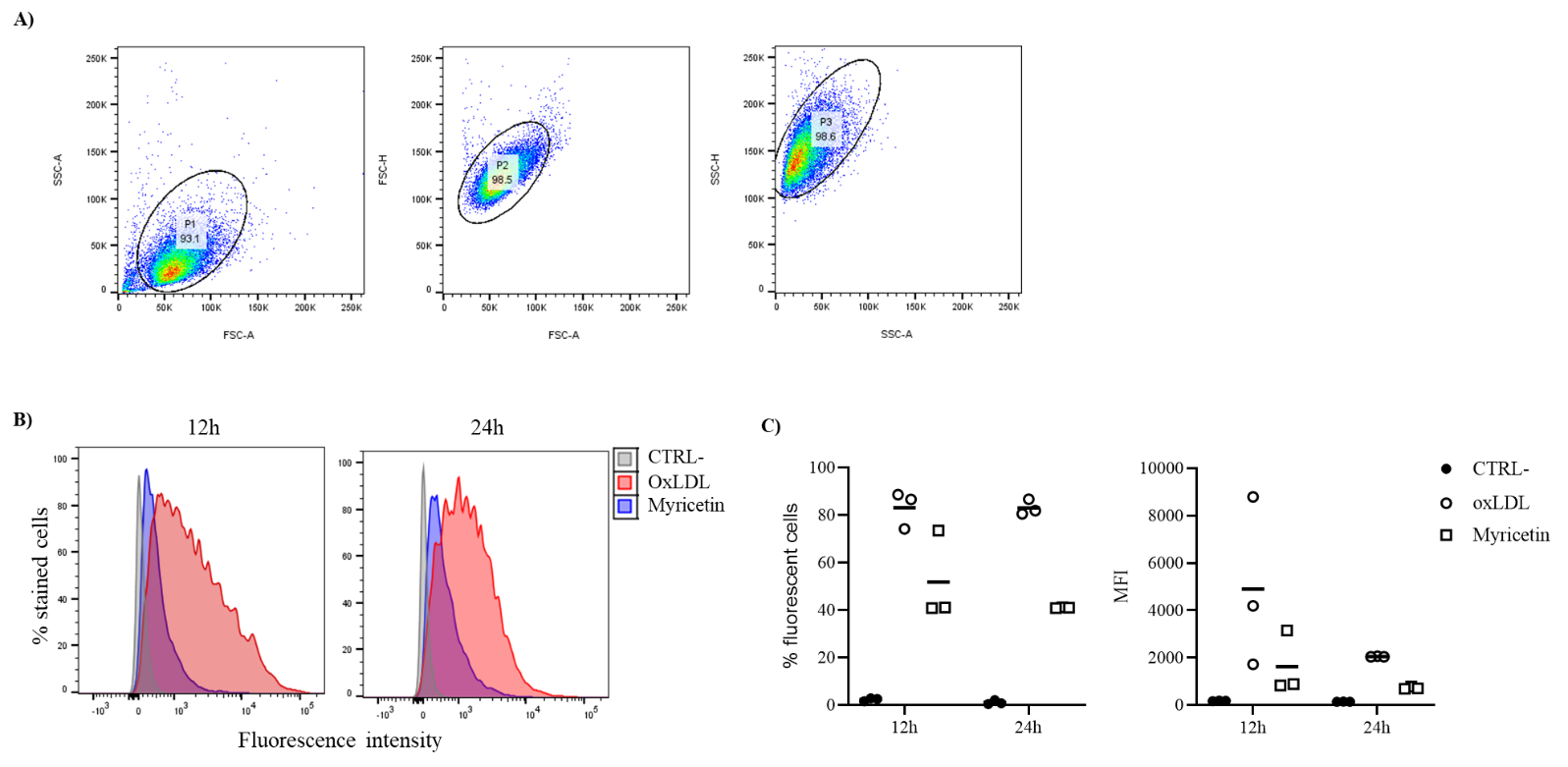


**Fig. S1. Assessment of the cholesterol uptake by MLC.** (A) Gating strategy applied for fluorescence-activated cell sorting (FACS). Representative forward (FSC-A) vs side light scatter plot (SSC-A) of untreated MLC for the selection of population of interest (left panel). FSC-H versus FSC-A plot (middle panel) and SSC-H versus SSC-A plot (right panel) for the exclusion of multiplets. (B-C) FACS analysis of MLC untreated (CTRL-, grey) or treated with OxLDL for 12h and 24h alone as technical positive control (OxLDL, red), or with the CD36 inhibitor myricetin 25µM 2h prior OxLDL (Myrecitin, blue): (B) histogram plots of y: percentage of stained cells normalized by mode vs x: fluorescence intensity; (C) mean value of the percentage of fluorescent cells per total events and mean fluorescence intensity (MFI). Individual data values are provided in an additional excel file (Additional file 2_Supporting data values).

**
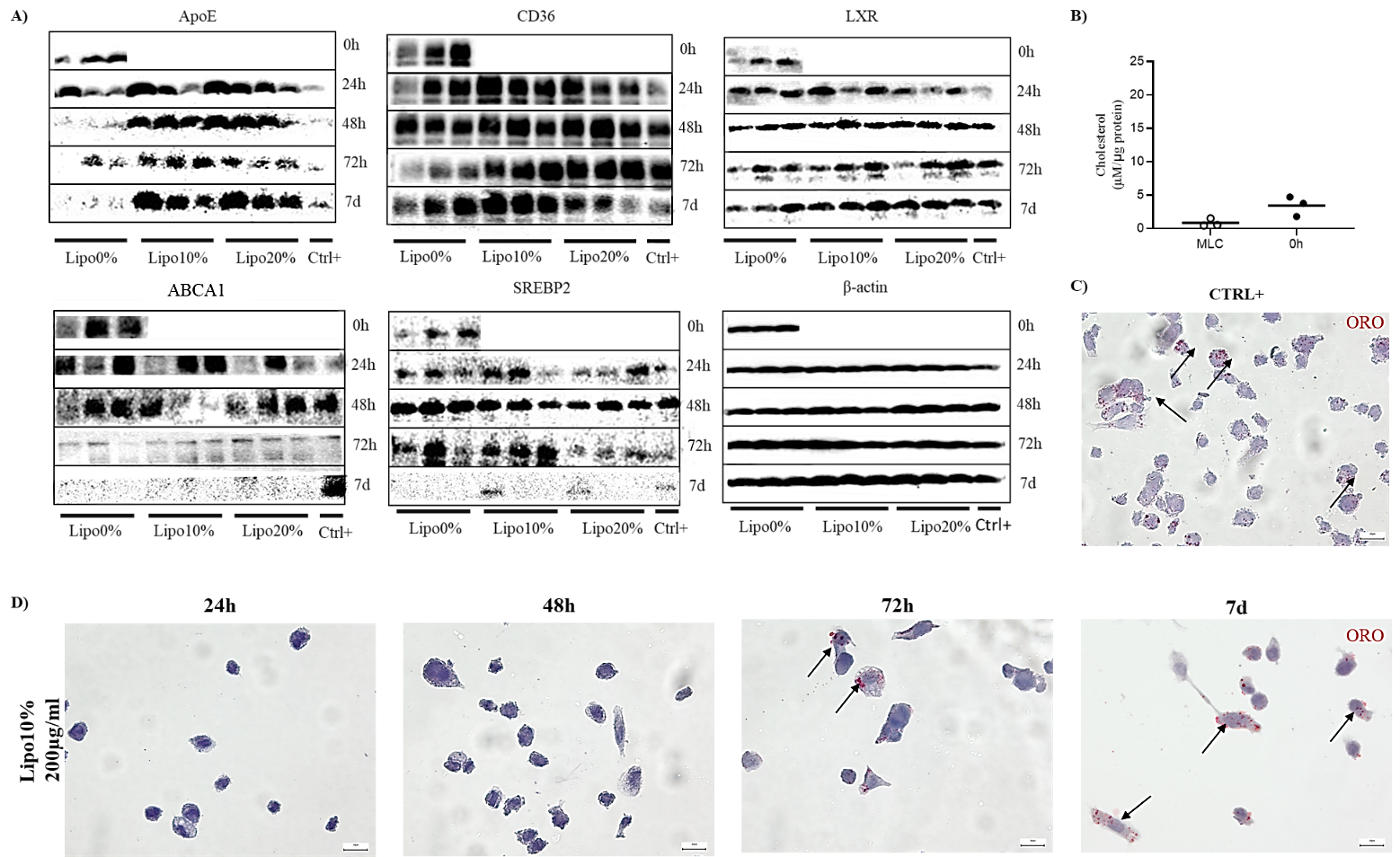
Fig. S2**

**Fig. S2.** **Metabolism of cholesterol-enriched liposomes and foam cells formation in MLC.** A) Western blot analyses for proteins of the RCT pathway and β-actin in cell lysate of MLC incubated with cholesterol-enriched liposomes 200µg/ml at 0% (Lipo0%), 10% (Lipo10%) or 20% (Lipo20%) of cholesterol for t 0h, 24h, 48h, 72h or 7days (7d). (B) Fluorometric cholesterol quantification of MLC untreated (MLC) or treated with CC at 1mg/ml (CC) for 0h. (C-D) Detection of foam cells. Oil Red O (ORO) staining of MLC treated with (C) OxLDL 50µg/ml for 24h as technical positive control or with (B) cholesterol-enriched liposomes 200µg/ml at 10% cholesterol (Lipo10%) for 24h, 48h, 72h or 7days (7d). Intracellular neutral lipid droplets are stained in red; cells are counterstained with Mayer’s hematoxylin (blue); foam cells are indicated by black arrows. Individual data values are provided in an additional excel file (Additional file 2_Supporting data values).

**Fig. S3**


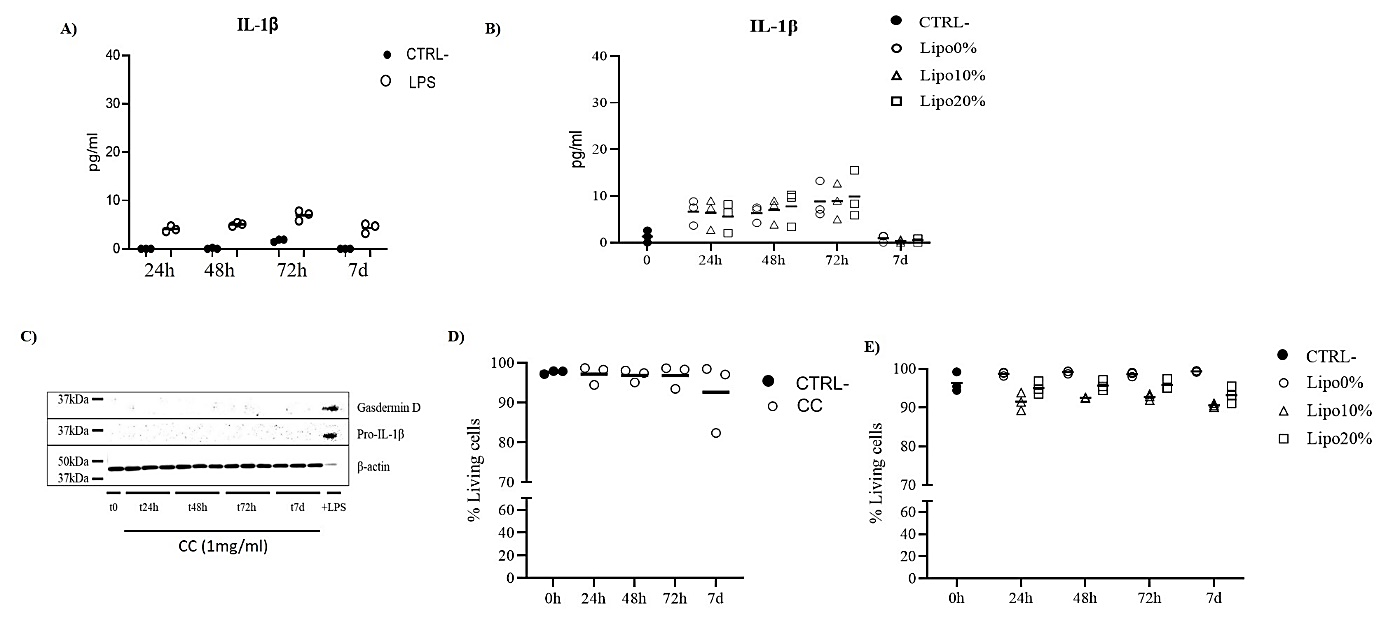


**Fig. S3. NLRP3 inflammasome analysis and viability in MLC.** (A) ELISA analysis of IL-1β in supernatant of MLC untreated (CTRL-) or treated with LPS 16.7µg/ml with a final endotoxin concentration of at 0.05EU (LPS). Data are shown as mean of three technical replicates. (B) ELISA analysis of IL-1β in supernatant of MLC treated with liposomes 200µg/ml at 0% (Lipo0%), 10% (Lipo10%) or 20% (Lipo20%) of cholesterol for t 0h, 24h, 48h, 72h or 7days (7d). (C) Representative results of western blot analyses for pro-IL-1β and gasdermin D in cell lysate of MLC incubated with CC 1mg/ml for t 0h, 24h, 48h, 72h or 7days (7d). MLC treated with LPS 5µg/ml for 3h (+LPS), served as positive control for antibody specificity validation. (D-E) Viability test in MLC treated with (D) CC or with (E) liposomes 200µg/ml at 0% (Lipo0%), 10% (Lipo10%) or 20% (Lipo20%) of cholesterol. Data are shown as mean of three independent experiments in different plates with three different cell passages and freshly prepared stimuli (N=3). Individual data values are provided in an additional excel file (Additional file 2_Supporting data values).


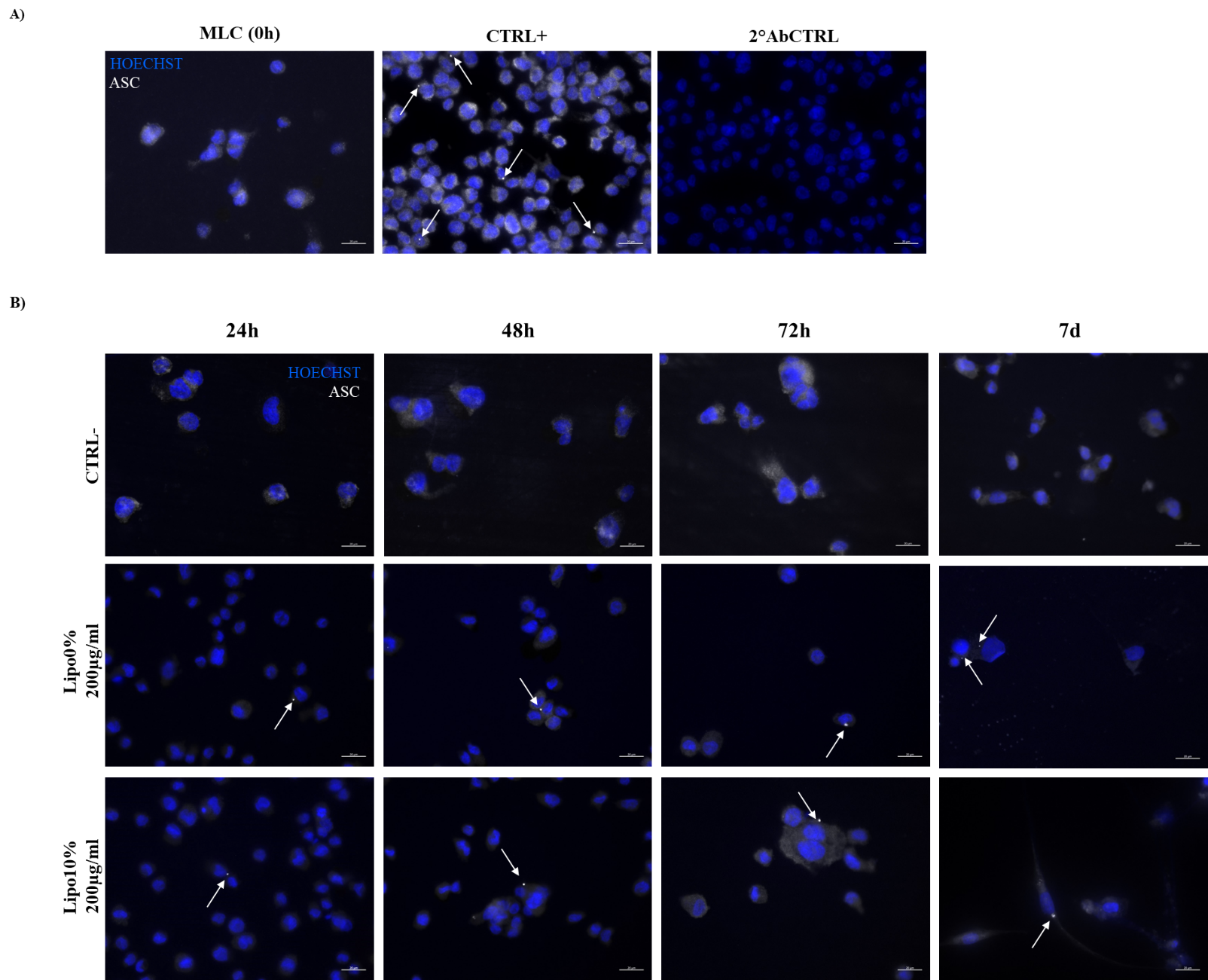
 **Fig. S4**

**Fig. S4.** **ASC-speck detection in:** (A) MLC untreated at t0h (MLC), treated with LPS 5µg/ml for 3h, serving as technical positive control for the method validation (CTRL+). As a negative control for the staining, cells were incubated with the secondary antibodies alone (2ndAbCTRL). (B) MLC untreated (CTRL-), or incubated with with cholesterol-enriched liposomes 200µg/ml at 0% (Lipo0%) or 10% (Lipo10%) of cholesterol for t 24h, 48h, 72h or 7days (7d). Cells were fixed and stained with anti-ASC antibody, followed by staining with PE-coniugated secondary antibody (false colored in white). Nuclei were stained by incubation with Hoechst 34580 (colored in blue). ASC speck are indicated by white arrows. All the micrographs were taken at the same magnification and reported with the same scale (scale bar = 20µm).
