# Supplementary material for: Cholesterol-mediated inflammation activation in alveolar macrophages
Source: BMC Biol. 2025 Dec 22;23:369. doi: 10.1186/s12915-025-02494-3 (PMC12750553; doi:10.1186/s12915-025-02494-3)

**Figure 1E,F – MLC+CC**

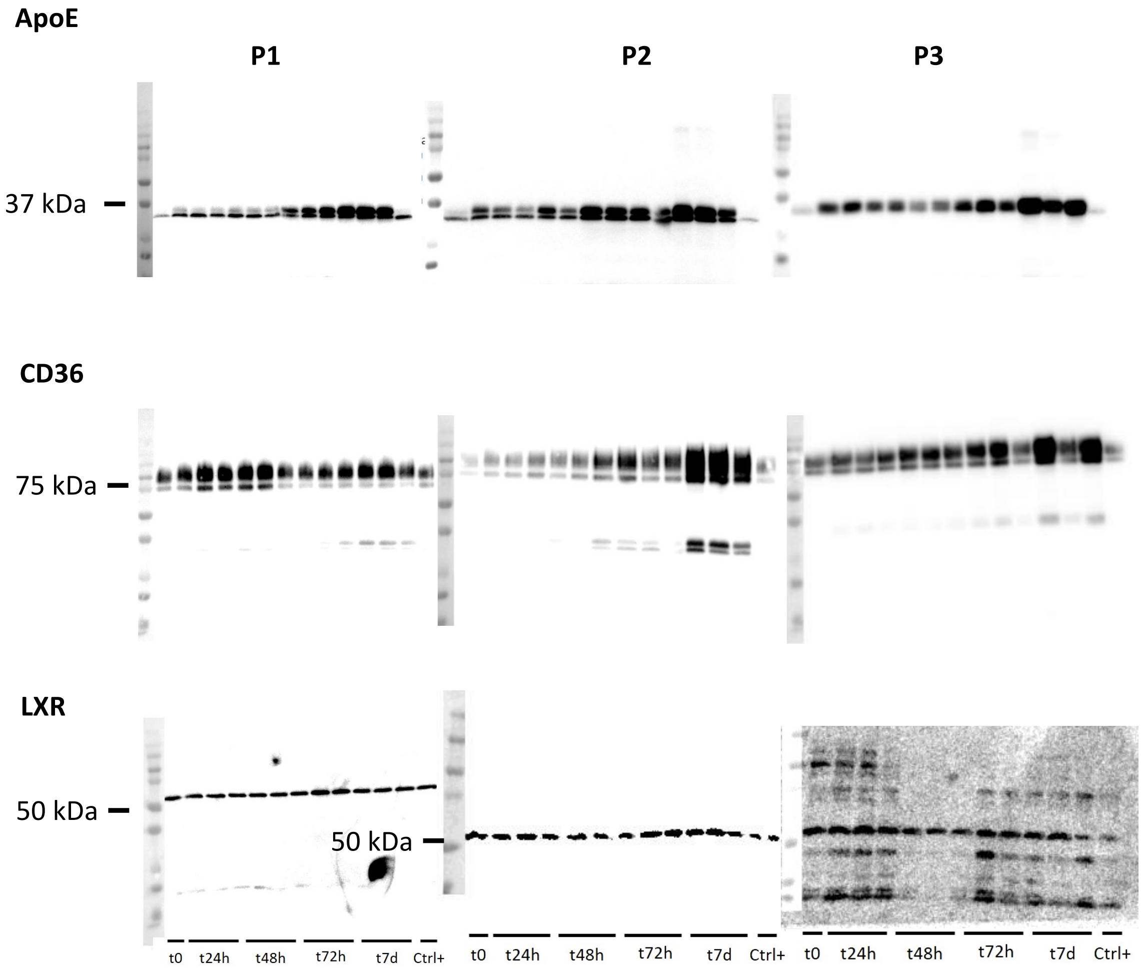

**Figure 1E,F – MLC+CC**

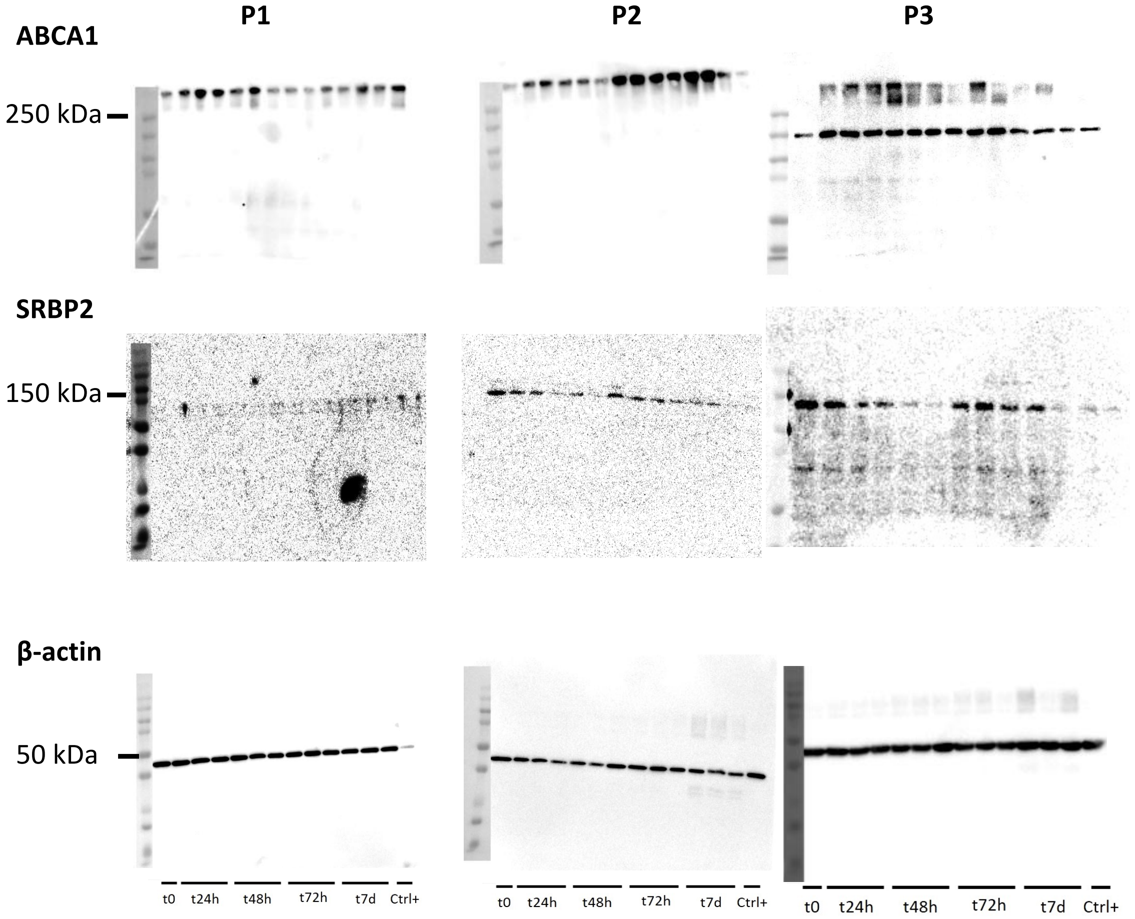

**Figure 1G,H , S2 – MLC+Liposomes**

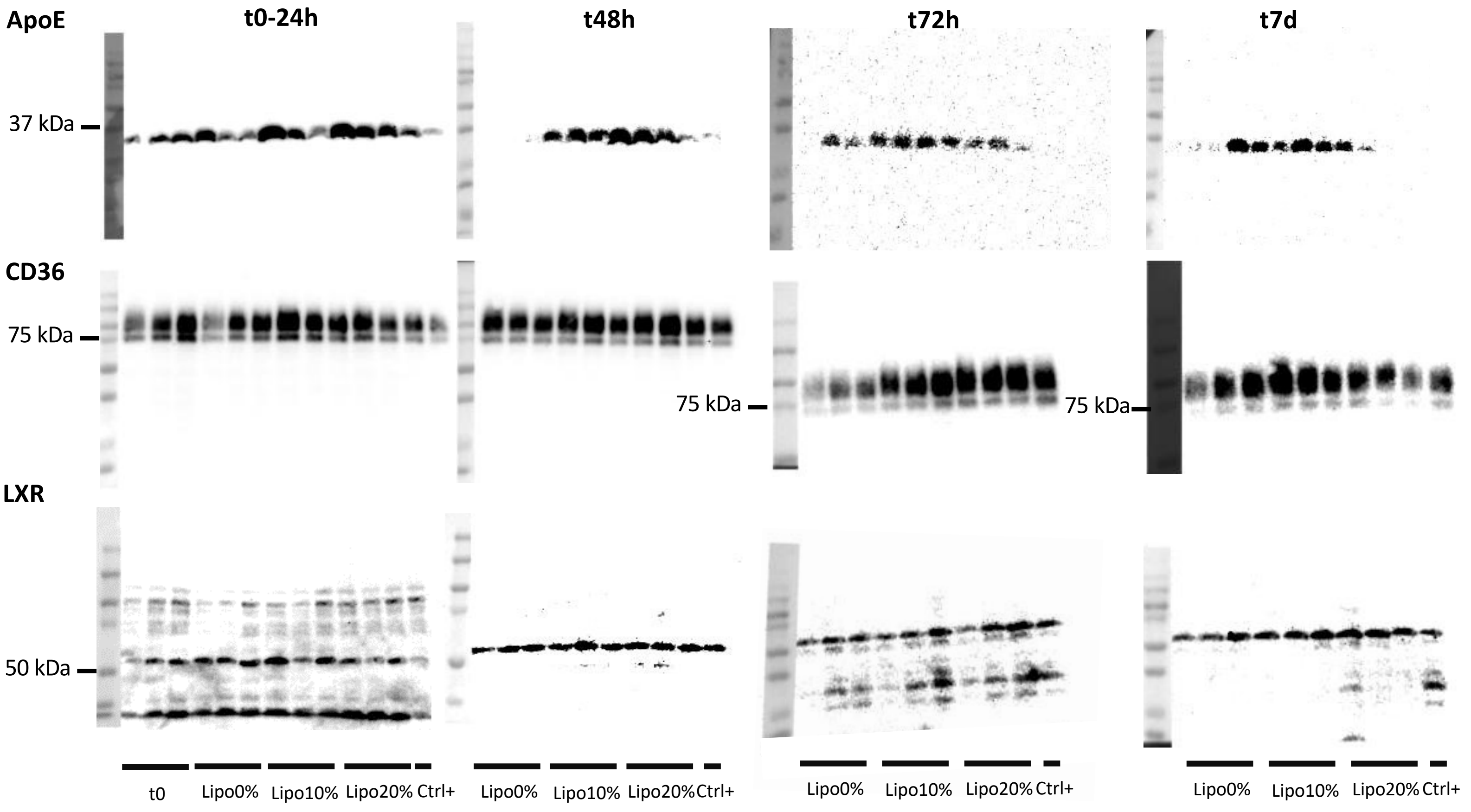

**Figure 1G,H, S2 – MLC+Liposomes**

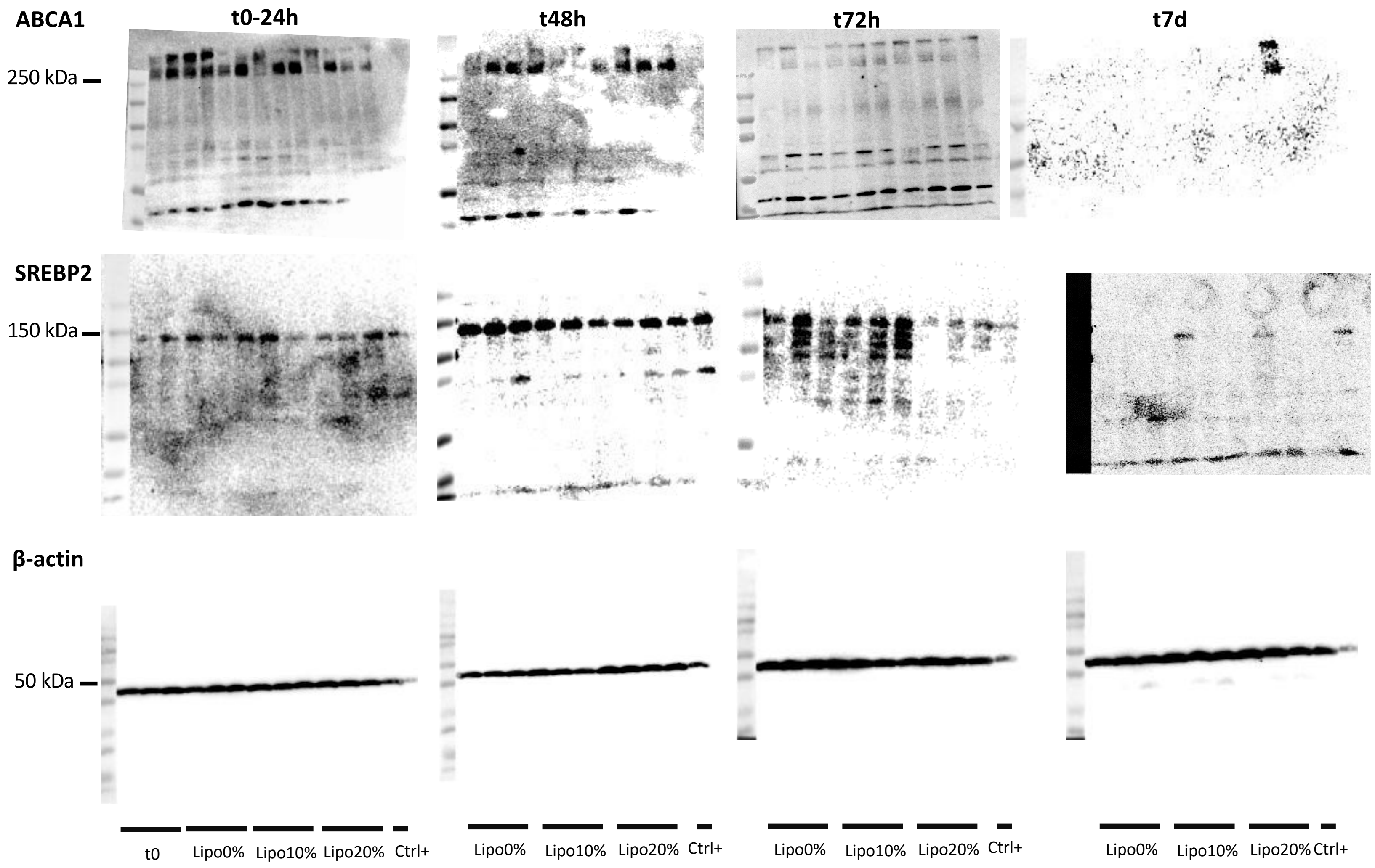

**Figure 4C – AM**

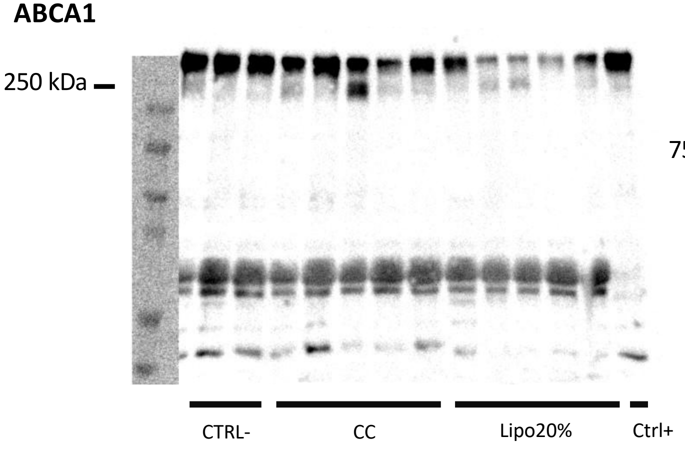

ABCA1
250 kDa
CTRL-
CC
Lipo20%
Ctrl+


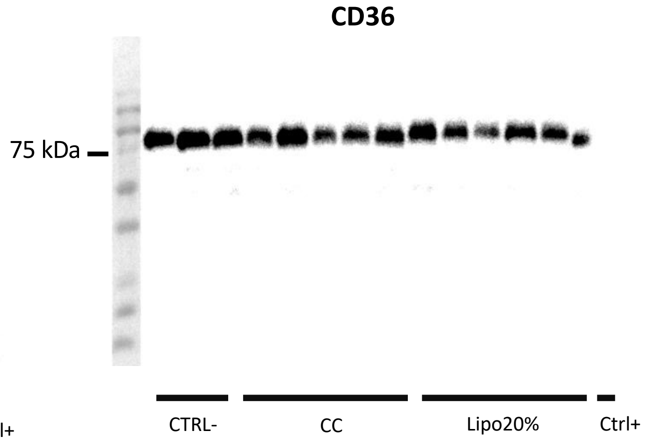

CD36
75 kDa
CTRL-
CC
Lipo20%
Ctrl+


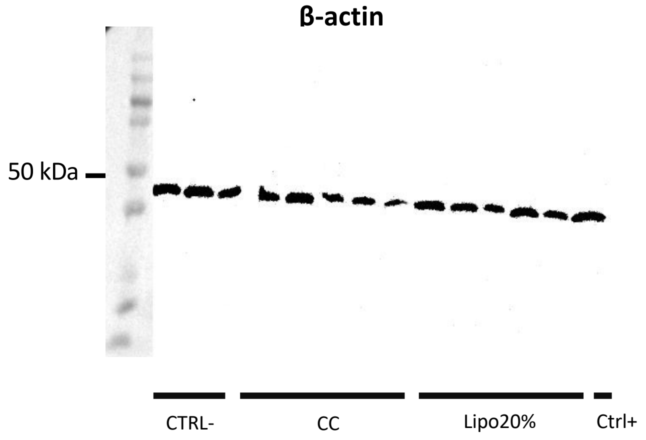

ß-actin
50 kDa
CTRL-
CC
Lipo20%
Ctrl+

**Figure S3 – MLC+CC**

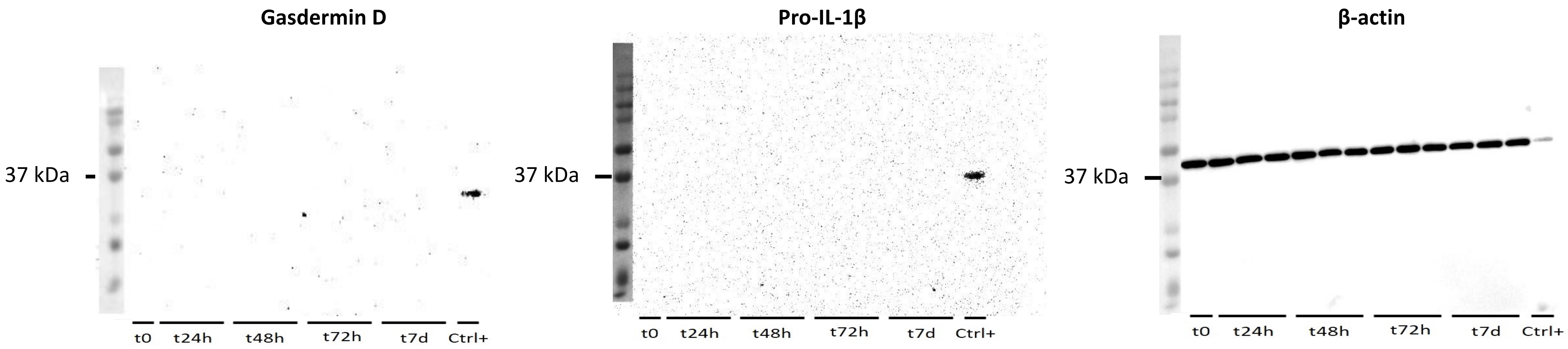

Supplement: Supplementary file 2 — Additional file 2. Supporting data values: Figs. 1–5 and Figures S1–S3 (https://zenodo.org/records/17929307). [file 12915_2025_2494_MOESM2_ESM.pdf]
