# Supplementary material for: Cholesterol-mediated inflammation activation in alveolar macrophages
Source: BMC Biol. 2025 Dec 22;23:369. doi: 10.1186/s12915-025-02494-3 (PMC12750553; doi:10.1186/s12915-025-02494-3)
Supplement: Supplementary file 3 — Additional file 3. Tables S1 and S2. Table S1 List of primary antibodies used for WB and/or immunofluorescence analysis. Table S2 List of secondary antibodies used for WB and/or immunofluorescence analysis [file 12915_2025_2494_MOESM3_ESM.docx]

**Supplementary data**

**Cholesterol-mediated inflammation activation in alveolar macrophages**

Sonia Giambelluca^1,2^, Matthias Ochs^1,2^, Elena Lopez-Rodriguez^1,2*^

^1^ Institute of Functional Anatomy, Charité - Univeristätsmedizin Berlin, Berlin, Germany

^2^ German Center for Lung Research (DZL), Berlin, Germany

* Correspondence:

Elena Lopez-Rodriguez: [elena.lopez-rodriguez@charite.de](mailto:elena.lopez-rodriguez@charite.de)

**Supplementary tables**

**Tables S1 –** List of primary antibodies used for WB and/or immunofluorescence analysis

| **Primary antibodies** | **Supplier** | **Catalogue #** | **Species reactivity** | **Concentration used** |
| --- | --- | --- | --- | --- |
| ABCA1 | Abcam | ab18180 | H, M | 1µg/mL |
| ApoE | Invitrogen | PA5-27088 | H | 0.7µg/mL |
| ASC/TMS1 | Cell Signalling Technology | 13833 | H | 0.8µg/mL |
| ASC/TMS1 | Cell Signalling Technology | 67824 | M | 0.1µg/mL |
| CD36 | Abcam | ab252922 | H | 0.5µg/mL |
| CD36 | R&D systems | AF2519 | M | 0.1µg/mL |
| IL-1β | Abcam | ab216995 | H | 0.5µg/mL |
| LXRα+β | Abcam | ab24362 | H | 1µg/mL |
| SREBP2 | R&D systems | AF7119 | H | 2µg/mL |
| β-actin | Abcam | ab8229 | H, M | 0.25µg/mL |
| β-actin | Sigma-Aldrich | A2228 | H, M | 0.4µg/mL |

H=human, M=mouse

**Tables S2 –** List of secondary antibodies used for WB and/or immunofluorescence analysis

| **Secondary antibodies** | **Species** | **Supplier** | **Catalog #** |
| --- | --- | --- | --- |
| HRP conjugated anti-rabbit | Swine | Agilent DAKO | P0399 |
| HRP conjugated anti-mouse | Rabbit | Abcam | ab6728 |
| HRP conjugated anti-goat | Donkey | Agilent DAKO | P0449 |
| PE coniugated anti-rabbit | Goat | Cell signalling technology | 79408 |
